# Evidence for sponges as sister to all other animals from partitioned phylogenomics with mixture models and recoding

**DOI:** 10.1038/s41467-021-22074-7

**Published:** 2021-03-19

**Authors:** Anthony K. Redmond, Aoife McLysaght

**Affiliations:** Smurfit Institute of Genetics, Trinity College Dublin, Dublin, Ireland

**Keywords:** Phylogeny, Molecular evolution, Phylogenetics

## Abstract

Resolving the relationships between the major lineages in the animal tree of life is necessary to understand the origin and evolution of key animal traits. Sponges, characterized by their simple body plan, were traditionally considered the sister group of all other animal lineages, implying a gradual increase in animal complexity from unicellularity to complex multicellularity. However, the availability of genomic data has sparked tremendous controversy as some phylogenomic studies support comb jellies taking this position, requiring secondary loss or independent origins of complex traits. Here we show that incorporating site-heterogeneous mixture models and recoding into partitioned phylogenomics alleviates systematic errors that hamper commonly-applied phylogenetic models. Testing on real datasets, we show a great improvement in model-fit that attenuates branching artefacts induced by systematic error. We reanalyse key datasets and show that partitioned phylogenomics does not support comb jellies as sister to other animals at either the supermatrix or partition-specific level.

## Abstract (web summary)

Branching artefacts can confound the reconstruction of deep evolutionary relationships. Here, the authors show improvement in partitioned phylogenomic analyses when using site-heterogeneous models and amino acid recoding, including in resolving the debated phylogenetic placement of comb jellies.

## Introduction

Porifera (sponges) are simple multicellular animals that lack both body symmetry and true tissues and organs, including a nervous, digestive and circulatory system^1,2^. Ctenophora (comb jellies) are comparatively more complex animals with rotational symmetry, that possess muscles, a through-gut, and a nervous system^1,2^. An active debate currently centres on which of these is the sister group to all other animals, obscuring our understanding of the evolutionary origins of key animal traits such as the nervous system and muscle^3–13^. The traditional view of animal phylogeny, with Porifera as the sister to other animals and Ctenophora as sister to Cnidaria, implies these key complex traits most likely originated once after the lineage divergence^2,8,14^. By contrast, positioning of Ctenophora as sister requires either their secondary loss in Porifera and Placozoa, or independent acquisition in Ctenophora and the ancestor of Bilateria and Cnidaria^3,6,11^. The Ctenophora-sister hypothesis first gained support from phylogenomic studies^3,6,15^ but is not supported under all data and modelling conditions^8–10,14,16,17^. While phylogenomics has revolutionized our understanding of the tree of life, commonly applied phylogenetic models do not account for a wide array of factors that are known to cause positively misleading phylogenetic estimates when overlooked^18,19^. Such factors include the non-independence of sites, variation of the substitution process across sites (site heterogeneity) and taxa (compositional bias), as well as site-specific variation over time of the substitution rate (heterotachy) and/or process (heteropecilly)^18^.

Substantial efforts have been made to account for many of these features, the most used, and perhaps most important^20^, probably being the development of the site-heterogeneous infinite mixture model CAT^21^ (as well as CATGTR, which infers an exchange matrix from the data rather than using flat, Poisson values). CAT/CATGTR offers improved resilience to long-branch attraction artefacts (LBA)^22^ and is almost always better fitting than standard site-homogeneous amino acid substitution models (e.g. WAG)^8–10,23–29^. This is because, by accommodating site-specific biochemical constraints, CAT/CATGTR can better detect saturation (i.e. multiple hidden substitutions)^8,20,22^, and therefore better identify cases of convergent evolution of identical amino acids in distantly related, fast-evolving species. By contrast, saturation and convergence are often underestimated when using standard site-homogeneous models, causing LBA^8,9,22^.

Another key approach that has gained traction recently is the use of amino acid recoding which groups the 20 amino acids into a smaller number of bins (usually 4 or 6 states), masking substitutions between biochemically similar and/or highly exchangeable amino acids^30,31^. Recoding is believed to reduce both saturation and compositional heterogeneity^30,31^, and possibly other biasing factors^32^, in amino acid datasets. In fact, when applied to resolve difficult phylogenetic questions, the level of heterogeneity present in recoded data can often be better accommodated by available models than that in the original amino acid data^9,24,33^. As such, recoding is thought to offer improved resilience to both LBA and compositional artefacts.

Despite having been designed to mitigate systematic errors, the performance of both CAT and recoding have come under scrutiny^34,35^. For example, it has been pointed out^11,12,34^ that standard phylogenomic analyses do not typically apply a single site-homogeneous model to an entire supermatrix, but rather partition datasets by genes, or by sets of genes (as merged under an optimality criterion)^36,37^, with each partition having a separate model. This makes relative-fit comparisons of CAT/CATGTR performance to that of a single site-homogeneous model (often carried out by cross-validation) unfair in these cases^11,12,34^, and so it can be argued that evolutionary relationships recovered only from CAT/CATGTR analyses may not in fact be favoured based on relative model fit^4,11,12,34,38,39^. Further, Whelan and Halanych^34^, have proposed that partitioning approaches and CATGTR are similarly accurate, with both outperforming CAT on simulated data, and have questioned whether the current implementation of the CAT model performs as intended. A recent simulation study has also cast doubt over the reliability of recoding^35^. The authors suggest that recoding performs poorly on highly saturated data (counter to expectations), and that the inherent loss of phylogenetic information surpasses the shielding recoding affords from compositional heterogeneity^35^. However, simulations often poorly imitate the heterogeneities present in real phylogenetic data, making the practical relevance of such findings unclear.

**Fig. 1:**
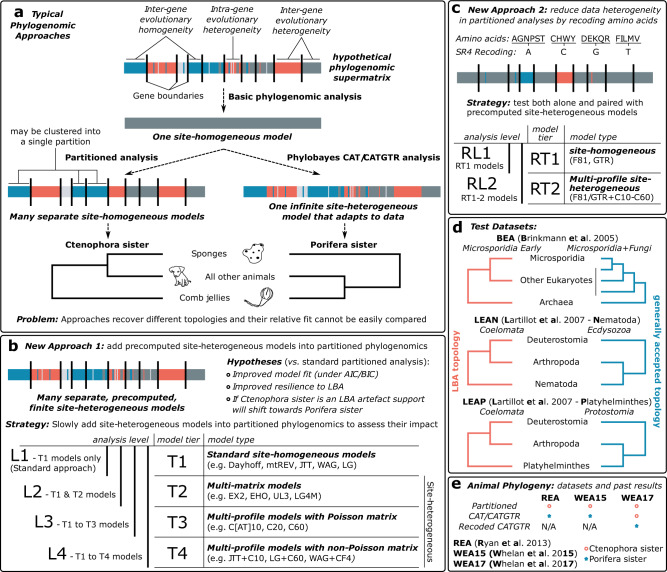
Overview of context and methodology of this study. **a** Summary of typical phylogenomic approaches: phylogenomics conventionally relies on a matrix of concatenated ortholog alignments (top) and thus includes different evolutionary patterns that exist both within and between genes (different colours indicate evolutionary heterogeneity). Despite this, basic phylogenomic analyses assume a uniform evolutionary process across the supermatrix (one site-homogeneous model). More sophisticated approaches may allow the evolutionary model to differ between genes (partitioned analysis, left: each gene is uniform, but inter-gene heterogeneity is accommodated – i.e. many site-homogeneous models), or use a single model where sites can vary infinitely and without respect to gene structure (right: infinite site-heterogeneous model). Relative model fit cannot easily be compared between these approaches, and, as illustrated for the animal phylogeny (bottom), they can produce conflicting results. To help resolve which is the correct animal phylogeny, we introduce site-heterogeneous models (allowing intra-gene variation) (**b**), and amino acid recoding (reducing data heterogeneity) (**c**), into partitioned phylogenomics, and lay out a strategy of comparing increasingly complex analysis levels with which to test whether this improves model fit and LBA resilience. We use three well characterized datasets with known susceptibility to LBA on which to test these approaches (**d**), before applying them to three key animal phylogeny datasets that have previously produced conflicting topologies under different modelling strategies (**e**).

The above methodological debate has been largely sparked by, and is deeply intertwined with, intense deliberation over which lineage is the sister group to all other animals: sponges, as has been thought since long before the availability of molecular data^8–10,14^; or fast-evolving comb jellies, for which support first emerged in the phylogenomic era^3,6,7,13,15,34,40^. Studies using partitioned site-homogeneous models have always recovered comb jellies as sister to other animals^3,4,6,7^. However, the branch leading to comb jellies is extremely long, and when re-analyses of the same datasets are performed using methods intended to alleviate systematic errors, such as site-heterogeneous models (i.e. CAT/CATGTR) and/or recoding, sponges tend to be recovered as sister to other animals (Fig. 1a)^8–10^. Analysis methods to assess underlying support in phylogenomic datasets at the level of individual sites and genes have also been devised with this problem in mind^40^. This approach has, conversely, revealed underlying support favouring Ctenophora sister^4,40^, but so far has only been performed under standard site-homogeneous models.

In the absence of fair relative-fit comparisons between CAT/CATGTR and partitioning, and with the appropriateness of recoding under debate, additional modelling or comparison approaches are required to help resolve which lineage is the sister group to other animals. Here, we incorporate both site-heterogeneous models (building upon the approach in ref. ^41^) and amino acid recoding into partitioned phylogenomics (Fig. 1b,c), and revisit the question of the branching order in the animal tree of life. To this end, we employ site-heterogeneous empirical mixture models, developed for single-gene analyses, for which relative-fit can be readily compared against standard site-homogeneous models using commonly applied optimality criteria (e.g. AIC, BIC; see Methods). We show the utility of these precomputed site-heterogeneous models to partitioned phylogenomics by comparing their relative-fit to standard site-homogeneous models for each partition, testing their relative ability to withstand LBA in a phylogenomic setting, and assessing their effect on underlying support in phylogenomic datasets. We also test the utility of amino acid recoding in partitioned phylogenomics using real phylogenomic datasets designed to exacerbate LBA (Fig. 1d), rather than potentially unrealistic simulations. Finally, we use this robust approach to re-analyse key datasets that have previously been used in attempts to resolve the root of the metazoan phylogeny (Fig. 1e). We provide novel evidence supporting sponges, and not comb jellies, as the sister group to all other animals. We conclude that contradictory results from previous studies were caused by LBA resulting from the use of poorly fitting, site-homogeneous models in partitioned phylogenomics.

## Results

### Testing the utility of mixture models and recoding in partitioned phylogenomics

To allow detailed exploration of the fit and phylogenetic influence of site-heterogeneous empirical mixture models to partitions we separated models into different model ‘tiers’ (T1–T4; Fig. 1b). T1 consists of standard site-homogeneous models only (e.g. JTT^42^, WAG^43^, LG^44^ etc.). T2 incorporates multi-matrix mixture models, which are designed to allow for sites with shared structural or rate properties to be modelled under different substitution matrices (UL2^45^, UL3^45^, EX2^45^, EX3^45^, EHO^45^, EX_EHO^46^, LG4M^47^). T3 incorporates precomputed site-heterogeneous profile mixture models, which are based on the infinite mixture model CAT (CAT10/20/30/40/50/60^48^; as well as CF4^49^), and T4 then combines the empirical CAT models with commonly used standard models, to provide precomputed models in the vein of the well performing CATGTR model (JTT/WAG/LG + CAT10/20/30/40/50/60; as well as JTT/WAG/LG + CF4)^27,50,51^. These model tiers were combined into increasingly complex analysis ‘levels’ (L1–L4); such that at L1 only the fit of T1 models is considered, while at L3, the fit of models from T1–T3 are considered (Fig. 1b). This testing of different tiers and levels would not be necessary for a standard analysis intending to use site-heterogeneous models in partitioned phylogenomics, where applying the more comprehensive L4 analysis would be most ideal. Our goal in doing so here was to explore the impact of different types of models on model fit and tree topology, as is sometimes performed in unpartitioned analyses^8,52,53^. Specifically, a consistent pattern can be generally expected to appear across the four analysis levels defined here—as model fit improves, so too should resilience to LBA. We believe this is an important consideration when tackling contentious and complex problems, such as whether sponges or comb jellies are the sister group to all other animals, as should such a pattern be apparent, then it arguably represents a much stronger line of evidence than support for one hypothesis over another under a single best-fitting model^54^.

We also incorporated an amino acid recoding strategy into our analyses and combined this with site-heterogeneous models (Fig. 1c), as applying recoding and CATGTR together has previously enabled a better absolute fit to real animal datasets to be obtained^9^. We applied SR4 recoding, a four-category scheme defined by Susko and Roger^31^, and set two recoded analysis levels (RL1 and RL2) in the same manner as for the amino acid datasets (Fig. 1c). The first considers the standard models F81 and GTR (model tier RT1), and the second considers both these models, as well as their pairing with the C10–C60 site-heterogeneous mixture models (e.g. SR4C10-F81, SR4C50-GTR; model tier RT2).

These modelling regimes were then tested on three real datasets designed to incite LBA when using standard site-homogeneous models. The first of these is a eukaryote dataset assembled by Brinkmann et al. (BEA)^55^ with Archaea as the outgroup. Standard analysis of this dataset incorrectly groups the fast-evolving Microsporidian, *Encephalitozoon cuniculi*, as sister to all other eukaryotes, rather than correctly placing them as close relatives to, or within (as deeply branching), fungi (Fig. 1d). This can be ascribed to a failure of standard site-homogeneous models to separate the long microsporidian branch from the long branch leading to the Archaeal lineages that make up the outgroup^55^. Discarding fast-evolving genes, improving taxon sampling, and employing a site-heterogeneous model (CAT or PMSF) have all been shown to correct this^55,56^.

The second and third datasets were generated by Lartillot et al. (2007)^22^ to test the resilience to LBA of CAT compared to standard site-homogeneous models. Both of these are bilaterian datasets and include Fungi as the only outgroup. These two datasets are differentiated by the presence of either nematodes (LEAN [i.e. Lartillot et al. Nematoda] dataset) or platyhelminths (LEAP) (Fig. 1d)^22^. The distant fungal outgroup is known to draw the long-branching nematode or platyhelminth clades toward the tree root when using standard site-homogeneous models, leading to high support for a spurious Coelomata clade (Arthropoda + Deuterostomia)^22^, and therefore is a suitable dataset to examine methods of mitigating LBA. Inclusion of closer outgroups, improving ingroup sampling, and use of site-heterogeneous models (CAT or PMSF), have all been shown to rectify this, favouring the monophyly of Protostomia (in the form of Arthropoda+Platyhelminthes), and of its subclade Ecdysozoa (Arthropoda+Nematoda)^22,56–58^.

### Site-heterogeneous mixture models and recoding improve model fit in partitioned phylogenomics

**Fig. 2:**
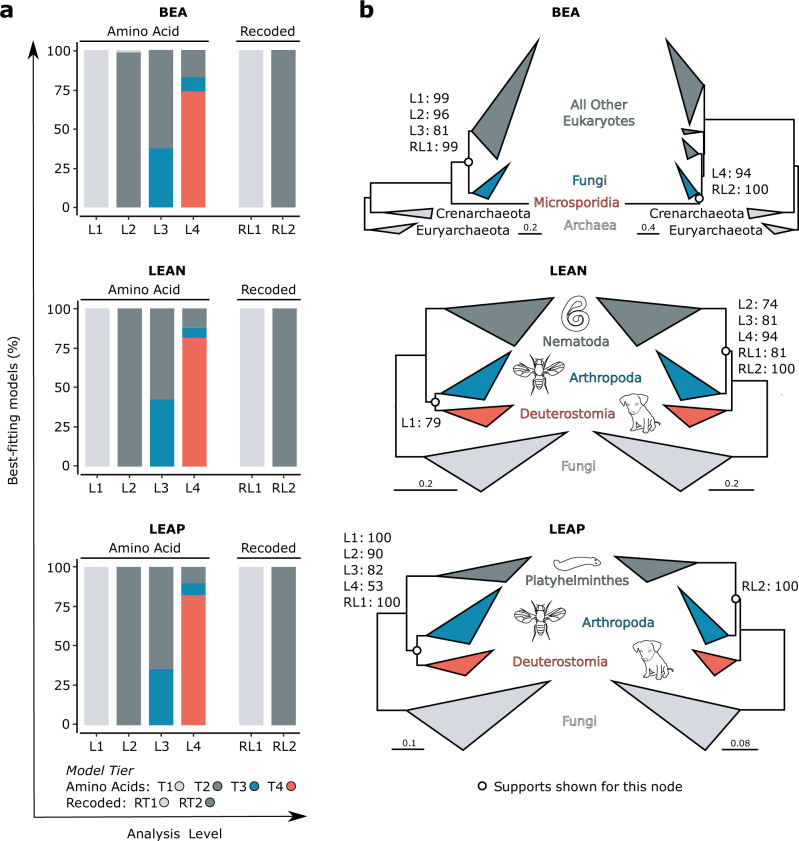
Analyses of test datasets incorporating site-heterogeneous models and SR4 recoding into partitioned phylogenomics. **a** Percentage of gene partitions best fit by each model tier (indicated by shading) at each analysis level for amino acid (left) and recoded (right) data. **b** Topologies recovered at different analysis levels for both amino acid (L1-4) and recoded (RL1-2) data in long-branch attraction tests. Ultrafast bootstrap (UFBOOT) percentages for the topology-defining node (indicated with a circle) are show for each analysis level. Scale bars are shown for each tree. For full tree topologies and UFBOOT support values see Supplementary Figs. 2-7.

Re-analyses of the three test datasets incorporating site-heterogeneous empirical mixture models in partitioned phylogenomics shows a remarkable change in model fit (that is, at analysis levels L2–L4, as L1 only includes standard site-homogeneous models). By L2 we observe that 131 of 133 gene partitions in the BEA dataset, and all 146 gene partitions in both the LEAN and LEAP datasets, are better fit (according to the Bayesian information criterion, BIC; see methods for rationale) by a site-heterogeneous model (Fig. 2a). As T3 and T4 models are incorporated at L3 and L4, not only are all genes in all datasets better fit by a site-heterogeneous model, but almost all partitions are also even better fit again than at lower levels, such that complex models from T4 appear to be most prevalently better fitting across all three datasets (BEA = 83%, LEAN = 88%, LEAP = 90%) (Fig. 2a). Exemplifying this, two of the most complex models applied, LG+C60 and WAG+C60, are best fitting for more than half of the genes in each dataset (BEA = 52%, LEAN = 61%, LEAP = 64%) (Supplementary Fig. 1). These findings (i.e. the highest possible number of site categories, 60, is often best fitting) indicate that substantial intra-gene heterogeneity, capable of influencing phylogenetic analysis, is likely typical in real data.

SR4-recoded datasets followed a similar pattern, with site-heterogeneous models (RT2 models) providing a better fit to all genes in all datasets than standard site-homogeneous models at RL2 (Fig. 2a). However, at odds with the improvement observed when complex exchangeability matrices (e.g. WAG, LG) are paired with multi-profile models in the amino acid analyses (i.e. at L4), combining GTR exchangeabilities with multi-profile models to analyse SR4-recoded genes never provides a better fit than simple F81 exchangeabilities. This indicates that this pairing is likely overfitting for short partitions, or single genes, when the data are simplified by SR4 recoding. In all, these results imply that phylogenetically relevant intra-gene site heterogeneity may be ubiquitous, or nearly so, in phylogenomic datasets, and starkly show that standard, site-homogeneous partitioned phylogenomic modelling approaches are overly simplistic.

### Site-heterogeneous mixture models and recoding attenuate LBA in partitioned phylogenomics

Phylogenomic analyses from L1–L3 for the BEA dataset show declining support for microsporidia as sister to all other eukaryotes as better-fitting site-heterogeneous models are applied (UFBOOT: L1 = 99%, L2 = 96%, L3 = 81%; Fig. 2b, Supplementary Fig. 2), until the best-fitting models are applied at L4, where the LBA topology is not recovered. Instead, Microsporidia are correctly nested within eukaryotes as sister to (or as deeply branching) fungi (UFBOOT: L4 = 94%; Fig. 2b, Supplementary Fig. 2). When the data are SR4 recoded the LBA topology is recovered only under standard models (UFBOOT: RL1 = 99%), while the (generally) accepted topology is recovered when better-fitting site-heterogeneous models are incorporated into the analysis (UFBOOT: RL2 = 100%; Fig. 2b, Supplementary Fig. 3).

Similarly, amino acid and SR4-recoded re-analyses of the LEAN and LEAP datasets reveal a clear pattern of declining support for the LBA topology (i.e. Coelomata) as better-fitting models are incorporated. For the LEAN dataset, the LBA topology is only recovered at L1 (UFBOOT: L1 = 79%), while the accepted topology uniting ecdysozoans in a clade is recovered with progressively higher support as model fit improves from L2–L4 (UFBOOT: L2 = 74%, L3 = 81%, L4 = 94%; Fig. 2b, Supplementary Fig. 4). SR4-recoded analyses never recover the LBA topology for this dataset, but the accepted topology is recovered with greater support when better-fitting mixture models are applied (UFBOOT: RL1 = 81%; RL2 = 100%; Fig. 2b, Supplementary Fig. 5). For the LEAP dataset, the accepted topology was not recovered even at L4 of the amino acid analysis; however, support for the LBA topology progressively decreases as model fit improves from L1–L4 (UFBOOT: L1 = 100%, L2 = 90%, L3 = 82%, L4 = 53%; Fig. 2b, Supplementary Fig. 6). The SR4-recoded dataset also recovers the LBA topology when standard site-homogeneous models are used (UFBOOT: RL1 = 100%); however, when better-fitting site-heterogeneous models are applied the accepted topology is maximally supported (UFBOOT: RL2 = 100%; Fig. 2b, Supplementary Fig. 7). Although LEAP and LEAN are derived from the same base dataset, the increased difficulty in overcoming LBA in the LEAP dataset compared to LEAN is not surprising. This is because although both lineages are fast evolving, platyhelminths (LEAP) are more distantly related to arthropods than nematodes (LEAN), and hence their shared evolutionary history, and the internal branch joining these lineages, is shorter^57,58^. Thus, this finding is fully consistent with the view that better modelling strategies are required for more difficult phylogenetic problems.

These analyses were further supplemented by assessing the summed log-likelihoods of both the LBA and accepted topology with the best-fitting models at each analysis level for each dataset (Supplementary Table 1). The topology with the highest log-likelihood was always consistent with the ML consensus tree for each analysis level except for LEAP L4 where the accepted topology had a slightly higher log-likelihood than the LBA tree which the consensus narrowly favours (UFBOOT = 54%).

Taken together, these results indicate that standard partitioned phylogenomics is highly susceptible to LBA, but that both amino acid recoding and better-fitting site-heterogeneous models can mitigate LBA in partitioned phylogenomics. The RL2 findings also support recent efforts pairing recoding and site-heterogeneous models to resolve difficult, ancient nodes in animal phylogeny^5,9,24,33^, despite the erosion of data relevant to more shallow nodes.

### Standard partitioning approaches may be problematic in phylogenomics

In phylogenomics, partitioning is generally thought to offer an improvement over unpartitioned analyses by accommodating inter-gene evolutionary heterogeneity^38,59^. However, evidence for this has come from analyses relying entirely on standard site-homogeneous models. Thus, we sought to determine whether partitioning also offers an improvement when using site-heterogeneous models. Previous studies analysing the test datasets used here, but without partitioning, have found support for the accepted topology under a variety of site-heterogeneous models^22,56,60^. However, for a more direct comparison to our partitioned site-heterogeneous analyses, we performed unpartitioned analyses using the most frequently best-fitting model at a given analysis level to compare at which analysis levels the accepted topology is first recovered at between partitioned and unpartitioned analyses. First, our results confirm that when site-homogeneous models are used the LBA topology is recovered for all datasets regardless of whether partitioning is applied (Supplementary Figs. 8-10). Next, we found that the generally accepted topology and/or strong support for the accepted topology were recovered at lower analysis levels (i.e. more easily) than when partitioning was employed (Supplementary Figs. 8-10). Exemplifying this, strong support for the accepted topology was recovered at L3 for LEAN and LEAP and L4 for BEA in unpartitioned analyses, whereas in our partitioned analyses amino acid analyses of LEAP (L1–L4) never recovered the accepted topology for LEAP and strong support for the accepted topology was only recovered at RL2 for all datasets. Thus, although partitioning with site-heterogeneous models confers improved LBA resistance compared to both partitioned and basic unpartitioned analyses using site-homogeneous models, it is less effective than unpartitioned analyses using site-heterogeneous models.

These findings are consistent with recent evidence that accounting for site heterogeneity is more important than partitioning (i.e. gene heterogeneity) when inferring phylogenies^41^. Taken together with the routine recovery of models with large numbers of site categories (e.g. LG+C60) as best fitting for individual gene partitions in our analyses, a plausible explanation for the reduced efficiency of site-heterogeneous models to resist LBA when partitioned is that within-gene heterogeneity drastically outweighs that between genes. As such, the use of genes as partition units is not ideal when site-heterogeneous models (which inherently assign similar site profiles from different genes to a single ‘partition-like’ site category) are available, as the constraints enforced by partitioning lead to overparameterization. With this in mind, partitioned phylogenomic analysis methods often cluster multiple genes into single partitions with the aim of reducing parameters and improving model fit to the data. To explore this, additional analyses of the three test datasets were performed with genes clustered into multi-gene partitions using 20% relaxed hierarchical clustering^34,36,37^ (see Supplementary Table 2 for clustered partition scheme info). At all analysis levels this produced results similar to those obtained when partitioning by gene alone, offering no consistent improvement in LBA resilience, and proved less effective at suppressing LBA than unpartitioned site-heterogeneous analyses (Supplementary Figs. 11-16). In all, these results imply that partitioning (at least when using genes as the basic unit) may be a problematic strategy in phylogenomics, hindering the ability of site- heterogeneous models to resist LBA through overparameterization, without shielding fully against lumping errors through underparameterization when genes are erroneously clustered into larger partitions under site homogeneous models^61^.

### Partitioned phylogenomics does not support Ctenophora sister

Despite partitioning apparently attenuating the LBA resistance afforded for site-heterogeneous models, we sought to determine whether incorporating site-heterogeneous models would help distinguish whether previous support for Ctenophora sister found using partitioning and site-homogeneous models was due to LBA, but expecting any effect to be less pronounced than has previously been observed using site-heterogeneous models in the absence of partitioning.

Three datasets that were previously shown to support comb jellies as the sister group to all other animals under partitioned site-homogeneous models were reanalysed using partitioned site-heterogeneous models and recoding to determine their impact on the root of the animal phylogeny (Fig. 1e). Two of these, from Ryan et al.^3^ (REA dataset) and Whelan et al. (2015)^7^ (WEA15 dataset) supported Ctenophora sister under partitioned phylogenomics, but were found by Pisani and colleagues^8^ to support sponges as sister to other animals under CAT/CATGTR. The third dataset (WEA17; Whelan et al. 2017^4^), supported Ctenophora sister under both CATGTR and partitioned phylogenomics. Despite supporting Ctenophora sister under CATGTR, WEA17 was later shown to support Porifera sister when amino acid recoding strategies and CATGTR are combined, an approach reported to allow better accommodation of systematic error^9,24^.

**Fig. 3:**
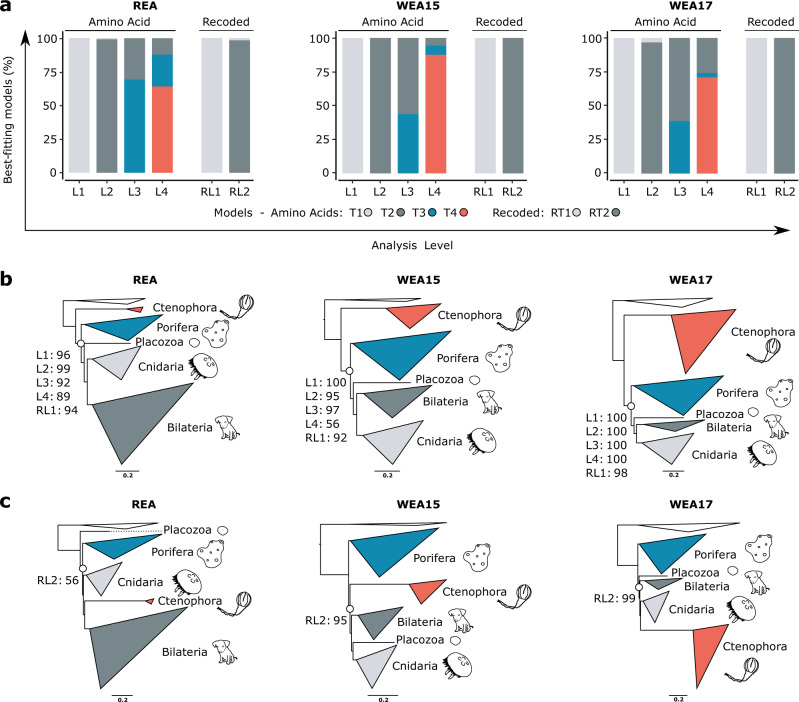
Analyses of test animal phylogeny datasets incorporating site-heterogeneous models and SR4 recoding into partitioned phylogenomics. **a** Percentage of gene partitions best fit by each model tier at each analysis level for amino acid and recoded data. Results are shown for each of the three datasets: REA, WEA15 and WEA17. **b** L4 topology recovered for each dataset, with UFBOOT support for Ctenophora sister (defining node indicated with a circle) under L1–L4 and RL1 shown. **c** Topologies recovered under RL2 for each dataset, with UFBOOT support for Placozoa sister shown for the REA dataset, and for Porifera sister for the WEA15 and WEA17 dataset. Note that for the REA and WEA17 datasets UFBOOT support for some backbone relationships are low under RL2 as discussed in the ‘Partitioned phylogenomics does not support Ctenophora-sister’ results subsection and Supplementary Note 1. For full tree topologies and UFBOOT support values see Supplementary Figs. 17-19 and 21-23.

Re-analyses of all three amino acid datasets recapitulated the results of the original studies at L1, providing maximal support for Ctenophora sister (Supplementary Figs. 17-19). However, we also found that almost every partition in every dataset was better fit by a site-heterogeneous model than by standard site-homogeneous models (Fig. 3a, Supplementary Fig. 20). When this is considered for the REA and WEA15 datasets, and applying the best-fitting models (i.e. at L4), partitioned phylogenomics no longer provides strong support for the placement of comb jellies as the sister group to all other animals (Fig. 3b, Supplementary Figs. 17 and 18). In fact, support for Ctenophora sister appears to be gradually reduced as model fit improves (i.e. from L1 to L4) (Fig. 3b, Supplementary Figs. 17 and 18). This weaker shift in support than observed in previous studies using unpartitioned CATGTR is as expected based on our analyses of the test datasets. On the other hand, support for Ctenophora sister is maximal even under the best-fitting models for the WEA17 dataset (Fig. 3b, Supplementary Fig. 19), in-line with CATGTR also recovering Ctenophora sister for this dataset.

When SR4 recoding is applied with standard models the Ctenophora-sister phylogeny is recovered for all three datasets (Fig. 3b, Supplementary Figs. 21-23). However, when better-fitting site-heterogeneous mixture models are incorporated at RL2 the REA dataset recovers Placozoa as sister to other animals, though support for this is very weak (UFBOOT = 52%). While both the WEA15 (UFBOOT = 95%) and WEA17 (UFBOOT = 99%) datasets recover Porifera sister with strong support (Fig. 3c, Supplementary Figs. 21-23). The placement of comb jellies is inconsistent between all three datasets, although support is not always strong, falling either as: sister to Bilateria (Acrosomata hypothesis^62^) in the REA dataset (UFBOOT = 43%); sister to Cnidaria (traditional, morphology-based Coelenterata hypothesis^2,14,63^) in the WEA17 dataset (UFBOOT = 38%); or sister to all non-sponge animals (recovered recently in some phylogenomic studies^8,9^) in the WEA15 dataset (UFBOOT = 95%) (Supplementary Figs. 21-23).

As such, and counter to previous studies, our results indicate that partitioned phylogenomics does not support Ctenophora sister. Instead, our results suggest that support for Ctenophora sister under partitioned phylogenomics reduces as better-fitting models are applied. This scenario is most consistent with Ctenophora sister being an artefact of model misspecification.

Although not our primary focus, other key nodes in the backbone animal phylogeny are also somewhat contentious, such as whether Xenacoelomorpha are sister to all other bilaterians^64,65^ or are sister to Ambulacraria^24,25,66,67^, and whether Placozoa are sister to both Bilateria and Cnidaria or to Cnidaria alone^33^. Interestingly, our re-analyses of the REA dataset (WEA15 and WEA17 do not include Xenacoelomorpha) show reducing support for Xenacoelomorpha as sister to all other bilaterian animals as better-fitting models are applied (REA dataset UFBOOT: L1 = 98%; L4 = 49%) (Supplementary Fig. 17), consistent with this placement being a systematic error^24^. We also recovered Placozoa as sister to Cnidaria in our recoded analyses of the WEA15 dataset (RL1 UFBOOT = 54%; RL2 UFBOOT = 90%) (Supplementary Fig. 22), in-line with the proposal that compositional heterogeneity obscures this relationship^33^.

Similarly, and more pertinent to the root of the animal phylogeny, some past studies have recovered support for sponge paraphyly at the base of the animal tree^8,68^. Such a scenario would provide compelling evidence for a sponge-like ancestral animal; however, in our analyses support for the monophyly of sponges generally tends to increase as better-fitting models are applied (Supplementary Figs. 17-19, 21-23) consistent with other analyses arguing against this hypothesis^7^.

### Partition-specific support for Ctenophora sister recedes under better-fitting models

**Fig. 4:**
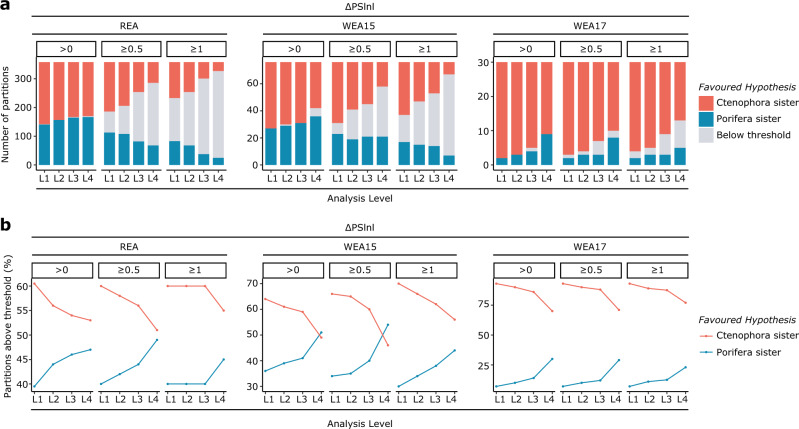
Partition-specific support for Ctenophora or Porifera sister under different analysis levels. **a** Number of partitions supporting either Ctenophora or Porifera sister from analysis levels L1–L4 at ΔPSlnl (change in partition-specific log-likelihood between trees/hypotheses) thresholds of >0, ≥0.5 and ≥1. **b** Relative proportion of partitions that support either Ctenophora or Porifera sister from L1–L4 (i.e. partitions that do not support either topology at that threshold level are not included).

To further explore which lineage is best supported as sister to all other animals we calculated the difference in partition-specific log-likelihoods (ΔPSlnl) under both the Ctenophora sister and Porifera-sister tree topologies for all three datasets at all four amino acid analysis levels. We categorized partitions into three increasingly stringent support brackets for each topology: 0.5 > ΔPSlnl > 0, 1 > ΔPSlnl ≥ 0.5 and ΔPSlnl ≥ 1 (see methods for further details). This strategy has previously shown support for Ctenophora sister, in the form of a greater proportion of sites and gene partitions favouring comb jellies over sponges as the sister group to other animals^4,40^. Until now the method has only been applied with standard models (i.e. L1), but our analyses incorporating site-heterogeneous mixture models (L2–L4) reveal that model choice can have a major impact on the results, with many partitions changing ΔPSlnl category and/or favoured hypotheses under different models (Supplementary Fig. 24). In-line with the improvement in model fit from L1 to L4, partition-specific log-likelihoods also improve from L1 to L4 assuming either Ctenophora or Porifera sister (Supplementary Fig. 25). Despite this, more partitions also favour Porifera sister over Ctenophora sister at L4 than at L1 for all datasets (Fig. 4a). Indeed, the proportion of partitions favouring Ctenophora sister generally decreases from L1 to L4, while the proportion of partitions favouring Porifera sister generally increases (Fig. 4b). These results are consistent with a similar analysis performed in a recent study^69^ and are also recapitulated when analysing recoded data at RL1 and RL2 (Supplementary Fig. 26).

Average ΔPSlnl values for each dataset appear to shift towards Porifera sister as better-fitting models are applied from L1 to L4 (Supplementary Fig. 27). However, the strength of ΔPSlnl values noticeably lowers from L1 to L4 (i.e. ΔPSlnl values less-strongly favour either hypothesis when better-fitting models are applied) (Fig. 4a), suggesting that much of the inferred phylogenetic signal favouring either hypothesis at L1 is non-historic and positively misleading (Fig. 4a; Supplementary Fig. 28; see Supplementary Note 1 for discussion of possible overfitting). This lowering of ΔPSlnl raises the possibility that the shift of average ΔPSlnl towards supporting Porifera sister may be an artefact of Ctenophora sister having greater initial support at L1 (Fig. 4a; Supplementary Fig. 28).

Contrary to this, we find that even when only partitions with a ΔPSlnl score ≥0.5 are considered, the same pattern of relative support emerges, with fewer partitions favouring Ctenophora sister and more supporting Porifera sister at higher analysis levels (Fig. 4). We also find that partitions supporting Ctenophora sister at L1 continue to do so less frequently at higher analysis levels than those supporting Porifera sister at L1 (Supplementary Fig. 29). Following this pattern, partitions supporting Ctenophora sister by ΔPSlnl ≥ 0.5 at L1 also less frequently continue to do so at higher analysis levels than those supporting Porifera sister by ΔPSlnl ≥ 0.5 (Supplementary Fig. 29). Partitions supporting Ctenophora sister by ΔPSlnl ≥ 0.5 at L1 also swap to supporting Porifera sister by ΔPSlnl ≥ 0.5 at L4 more frequently than those supporting Porifera sister by ΔPSlnl ≥ 0.5 at L1 swap to supporting Ctenophora sister by ΔPSlnl ≥ 0.5 at L4 (Supplementary Fig. 29).

In all, these results not only strongly indicate Ctenophora sister is a systematic error, but also reveal the influence of substitution model when estimating ‘phylogenetic’ signal from partition-specific log-likelihood values.

## Discussion

Understanding the effects of, and accounting for, systematic errors in phylogenomic analyses is necessary to resolve the tree of life^9,18,24,70^. Here, we have improved the ability of partitioned phylogenomics to address systematic errors, and hence recover more accurate phylogenies, by incorporating amino acid recoding and better-fitting site-heterogeneous mixture models. Utilizing this new approach, we discovered that partitioned phylogenomics does not support comb jellies as the sister group to all other animals, and that previous support for this topology derived from the use of poorly fitting models.

Several studies have already shown that gene family^8,71^ and unpartitioned phylogenomic analyses using more sophisticated substitution models^8–10^ reject Ctenophora sister in favour of Porifera sister. Here, we have consolidated these findings by directly showing that the primary remaining lines of evidence supporting Ctenophora sister, partitioned phylogenomics^4,7^ and measures of underlying support (such as ΔPSlnl values)^4,40^, do not do so when better-fitting site-heterogeneous models are incorporated into the analysis. Thus, the Ctenophora-sister hypothesis can now be wholly rejected in favour of the traditional Porifera-sister scenario of animal evolution, wherein the animal ancestor did not possess key traits such as a nervous system, muscles or a mouth and gut^8,10^.

**Fig. 5:**
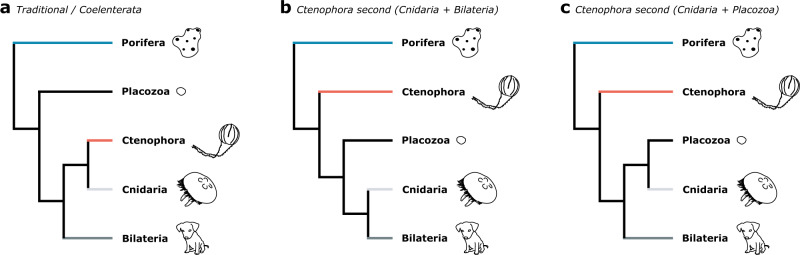
Remaining plausible hypotheses of the backbone animal phylogeny following rejection of Ctenophora sister in favour of Porifera sister. Ctenophora is now most likely placed either as sister to Cnidaria (Coelenterata) within the Eumetazoa, as is traditional (**a**), or as the sister group of all non-sponge animals (Ctenophora second). In this latter scenario it is not yet clear whether Bilateria (**b**) or Placozoa (**c**) would fall sister to Cnidaria.

Focus must now turn to another difficult problem, resolving Ctenophora’s position amongst the remaining animals. We see two plausible placements, the first of which is the traditional scenario as sister to Cnidaria (Coelenterata)^14^ (Fig. 5a), which has recently been recovered in morphological^63^ and gene family presence/absence^71^ analyses, as well as in preliminary efforts to eliminate heteropecilly in phylogenomics^10^. This scenario strongly implies shared origins of many complex animal traits between Cnidaria, Bilateria and Ctenophora (e.g. neural systems, muscle), followed by substantial modification and genomic divergence along the long branch leading to Ctenophora. The second placement is as sister to all non-sponge animal lineages (Fig. 5b). This latter ‘Ctenophora second’^8,10^ scenario has been reported in a number of phylogenomic studies attempting to account for compositional heterogeneity, but, like Ctenophora sister, does not lend itself to understanding whether key traits have evolved more than once due to the placement of Placozoa as sister to Cnidaria and Bilateria^10,12^ (Fig. 5b).

Interestingly, some recent studies have reported Placozoa as sister to Cnidaria (sometimes paired with Ctenophora second) when minimizing compositional heterogeneity^5,33^, and we have also recovered some support for this (RL2 analysis of WEA15 dataset) (Fig. 3c). We propose this topology (Fig. 5c) as a plausible variant of Ctenophora second, and given that fully independent acquisition of multiple, otherwise unique, traits in immediate sister groups seems highly unlikely, suggest that this supports a shared origin of (at least the building blocks of) numerous complex animal traits, including nervous systems and muscles. This topology fits well with the apparent mixture of shared and distinct features of the nervous system in Ctenophora compared to that of Cnidaria and Bilateria^3,6^ and implies lability early in nervous system evolution such that substantial post-speciation novelty arose and/or differential loss of ancestral components occurred. Such a scenario is not unprecedented and draws an interesting parallel to the early evolution of vertebrate adaptive immunity, with jawed and jawless vertebrates having a striking mix of shared and distinct genetic and cellular components^72–74^.

Sound rejection of any of these scenarios will require more sophisticated phylogenomic approaches that better account for the heterogeneity present in animal datasets than have been applied to the problem to date^9^. For example, ‘Ctenophora second’ has only been recovered from genetic studies and may yet go the way of Ctenophora sister and prove to be another (albeit less severe) artefact of model misspecification. Beyond modelling concerns, further complications to resolving the animal phylogeny warrant future consideration (e.g. incomplete lineage sorting and orthology errors due to hidden paralogy, contamination or horizontal gene transfer)^59,75^. Orthology errors have been shown previously for the REA and WEA15 datasets^10^, while the WEA17 dataset appears to have poor occupancy of Ctenophores and a very high level of orthology errors (Hervé Philippe, Personal Communication 2020; Jesus Lozano Fernandez, Personal Communication 2020), both of which can lead to phylogenomic error^76^. It is conceivable that these issues contribute to the stronger signal for Ctenophora sister in this dataset, and it is notable that Porifera sister was recovered when such errors were best accounted for previously^10^. Improving our understanding of the interplay between data and modelling errors will undoubtedly help to resolve the position of Ctenophora and Placozoa in the animal tree of life.

Our results revealed a striking, almost ubiquitous, improvement in model fit across all analysed datasets when site-heterogeneous models were considered, with complex, high dimensional T4 models (e.g. LG + C60) being better fitting for most genes/partitions (Figs. 2 and 3, Supplementary Figs. 1 and 20). This model-fit improvement is accompanied by improved resilience to long-branch attraction, suggesting that phylogenetically relevant intra-gene site-specific diversity of biochemical constraints is extensive in real datasets, and should be regularly considered in both phylogenomic, as is usually achieved with CAT/CATGTR, and gene tree analyses. In fact, this high prevalence of the most complex models tested here as best fitting for single genes implies that they may still be underfitting the data (see Supplementary Note 1 for a detailed exposition on under- and overfitting in this study), and that even more complex models (e.g. UDM models^77^), may yet prove better fitting and more resilient to LBA. Taking this probable underfitting of intra-gene site-diversity together with the apparent overfitting enforced by partitioning our results suggest that unpartitioned site-heterogeneous models are likely the best computationally tractable option currently available for concatenated phylogenomic analysis. Furthermore, the recurrent recovery of C60 based models (i.e. models with 60 site categories) as best fitting for single genes rebuts previous arguments^34,78^ that hundreds of site categories (as are often inferred by CAT/CATGTR) are not required to infer the root of the animal phylogeny in unpartitioned analyses. In-line with this Whelan and Halanych’s simulations questioning the reliability of the CAT model’s inference of large numbers of site categories (and its placement of Porifera as sister to other animals)^34^ were found to be flawed^41^, and improved simulations are instead consistent with Ctenophora sister deriving from LBA^79^.

Our results, and those of many previous empirical studies, also conflict with simulations by Hernandez and Ryan^35^ that suggest recoding is outperformed by amino acid analyses even when data are saturated or compositionally heterogeneous. It is noteworthy that recoding is usually applied with specific (long/ancient) branches in mind and that detrimental effects to other parts of the tree are to be expected (e.g. at shallower nodes) and may be safely overlooked when results are backed up by complementary analyses, such as obtaining consistent results from different recoding schemes^9^, or evidence from the use of site-heterogeneous models or partition-specific support at the amino acid level, as applied here. Nevertheless, a fuller understanding of the implications of recoding is needed, along with further development of more sophisticated recoding strategies and selection criteria^31^.

Understanding the source and strength of conflicting phylogenetic signals may be useful when tackling difficult-to-resolve evolutionary relationships. Quantifying and comparing support for alternative hypotheses for a given gene/site allows for dissection of the signal favouring one hypothesis or another in a dataset, and our results reject previous studies taking this approach that have found support for the Ctenophora-sister hypothesis^4,40^. Instead we find support for a shift from Ctenophora sister towards Porifera sister as better-fitting models are incorporated. Importantly, just as this approach can be used to identify outlier genes/sites that can change topologies in phylogenomic analyses^40,80^, our results also reveal that it can identify shifts in support (e.g. as better-fitting models are incorporated) even when they are below the point of perturbing bootstrap support, which are nonetheless clear and informative (e.g. WEA17 dataset). This strategy may provide a useful resource to future studies by enabling detection of LBA even when the most sophisticated models available are misled.

In summary, our study introduces new approaches that improve resistance to LBA in partitioned phylogenomics, highlighting the importance of accommodating site heterogeneity in phylogenetics, and provides conclusive evidence that Ctenophora sister is a phylogenomic artefact stemming from the use of overly simplistic models. Consternation over which lineage is sister to other animals has had a major influence on development of novel approaches and reassessment of the quality of commonly applied phylogenetic methodologies. This looks set to continue, with many avenues of research highlighted by this debate yet to be explored.

## Methods

### Datasets

The LEAP (35,373 sites, 146 genes and 32 taxa) and LEAN (35,373 sites, 146 genes and 37 taxa) datasets^22^ did not have gene partition information. As such, gene boundaries within the supermatrices, were estimated by manual inspection of the alignment for obvious starts/ends of genes, as well as BLAST^81^ searches against the NCBI nr protein database to identify/verify gene starts/ends. These identified gene boundaries were then used to specify gene partitions and as starting point for analyses clustering genes into larger partitions. This information was already available for the other test dataset BEA (24,294 sites, 133 genes and 40 taxa)^55^.

For the animal phylogeny analyses the REA (88,384 sites, 406 genes and 60 taxa; named’EST’ dataset in the original study)^3^ and WEA15 (23,680 sites, 89 genes, 62 taxa; named ‘dataset 16’ in the original study)^7^ datasets were analysed partitioned by gene. The rogue taxon *Xenoturbella bocki* was excluded in the case of the REA dataset, and both datasets were analysed including only the closest outgroup (choanoflagellates), following Pisani et al. (2015)^8^. Additional analyses at L1 and RL2 including *X. bocki* verified that its removal had a negligible impact on the root of the animal phylogeny (Supplementary Fig. 30). Relaxed clustering has previously shown that partitioning by gene is the best fit for the WEA15 dataset and this is the partitioning scheme that was used in the original study^7^. The WEA17 (49,388 sites, 117 genes in 31 partitions and 76 taxa; named ‘Metazoa_Choano_RCFV_strict’ in the original study)^4^ dataset was analysed using the 31 partitions from the initial analysis^4^, but the amino acid Level L4 and SR4-recoded Level RL2 analyses were also performed using the 117 genes as partitions and this did not change the relevant results (Supplementary Fig. 31). We decided not to analyse the more gene-rich dataset of Simion et al. (2017)^10^, as analyses at higher analysis levels were estimated to require >1000GB of RAM. We envision that future incorporation of the PMSF^56^ method into partitioned phylogenomics has the potential to reduce the memory and run time requirements of partitioned analyses using site-heterogeneous models, which will enable rapid analysis of large datasets.

### Model fitting and phylogenomics

All model testing and phylogenomic analyses were performed in IQ-tree (v. 1.5.4-omp, except for hierarchical clustering partition-finding analyses of test datasets and subsequent reanalyses with site-heterogeneous models and/or recoding which were performed with v. 1.6.12)^82–84^. Best-fit models were chosen according to the commonly applied Bayesian Information Criterion (BIC) in modelfinder^84^ (as packaged in IQ-tree) (-m TEST), as this has higher specificity and so should be more conservative when considering complex models than the other commonly applied method, the Akaike information criterion (AIC)^85^. However, we note that Multi-profile site-heterogeneous mixture models (C10–60) were specified with their precomputed weights, rather than optimizing these for the data, given that they were mainly applied to short single-gene partitions. As such L3 and L4 models have the same number of free parameters as ‘+G’ L1 models (as C10–C60 incorporate ‘+G’ already), for example Poisson+G, LG+G, C10 and LG+C60 all have the same number of parameters, meaning that the choice of information criteria is irrelevant. On the other hand, for multi-matrix L2 models this is not the case as each matrix adds an additional free parameter (e.g. UL3 [a 3 matrix model] has 2 extra parameters than LG, whereas EX_EHO [a 6 matrix model] has 5), which can be penalised in model selection. For recoded analyses the addition of C10–C60 also adds no free parameters and so only the choice of GTR (which has more free parameters) over F81 is penalised by BIC. Site-heterogeneous models were specified explicitly for inclusion in model testing and phylogenomic analyses (-madd). Four discrete gamma rate-heterogeneity categories were specified for every site-heterogeneous model (where not already incorporated in IQ-tree), while fit with and without rate heterogeneity across sites was tested for standard models (as is default in IQ-tree). Partitioned maximum likelihood phylogenetic analyses were performed allowing each partition to have its own evolutionary rate, but with linked branch lengths (-spp)^86^. Unpartitioned analyses were performed for the three test datasets using the most frequently best-fitting model for each analysis level until the generally accepted topology was recovered, after which point additional analysis levels were not considered necessary for comparison, particularly as these datasets have previously been analysed with unpartitioned site-heterogeneous models, including some of those used here. Where genes were merged into larger partitions this was initially performed using the 20% relaxed clustering approach (-m TESTMERGE -rcluster 20)^36,37^. For analyses at L2–L4, and at RL1 and RL2, the partitions inferred at L1 were applied for the purpose of comparison, rather than re-computing partitions at each level, which might be considered more appropriate. All branch supports were derived from 1000 ultrafast bootstrap (UFBOOT) replicates (-bb 1000)^87^. Ultrafast bootstrap is less conservatively biased than standard bootstrap, providing support percentages that are more directly interpretable^87^, and as such we interpret values ≥95% as providing strong support. We also tested the SH-aLRT^88^ and aBayes^89^ measures of branch support for the BEA dataset, but found these tests recovered less nuance than did ultrafast bootstrap (Supplementary Fig. 32). Summed log-likelihoods were calculated for the LBA and accepted topologies for each test dataset at all analysis levels partitioned by gene by using a fixed topology in IQ-tree (-te). The topology recovered at L1 was used to represent the LBA tree for each dataset, while for the accepted topology the tree recovered at L4 was used for BEA and LEAN, and the RL2 tree for LEAP. Partition-specific log-likelihoods for both Ctenophora sister and Porifera sister were also calculated in IQ-tree (-wpl) under fixed topologies. For these analyses the tree recovered in L4 analyses (for that dataset) was used as the fixed tree to calculate Ctenophora-sister log-likelihoods, given that this topology was recovered at L4 for all three datasets. As a fixed input tree with which to calculate Porifera sister log-likelihoods the L4 topology was modified to swap the branches leading to Porifera and Ctenophora, such that the resultant tree placed Porifera as sister to all other animals and Ctenophora as sister to all remaining animals (i.e. excluding Porifera). For these analyses only those partitions with at least one choanoflagellate (i.e. outgroup), one sponge, one comb jelly and one other animal sequence were considered for plotting and downstream analyses as non-zero ΔPSlnl values calculated for other partitions must be analytical artefacts^90^. ΔPSlnl values were calculated by simply subtracting the PSlnl value under Porifera sister from that under Ctenophora sister for each partition in each dataset. To categorize support thresholds in favour of one topology over the other with which to examine the ΔPSlnl data we specified three levels of permissiveness. The first only requiring ΔPSlnl > 0 was maximally permissive, followed by the more stringent threshold values of ΔPSlnl ≥ 0.5 (as previously used by Shen et al. (2017)^40^ at the site level), and ΔPSlnl ≥ 1 (twice the previous stringency). Despite this, it must be noted that higher ΔPSlnl values may be required to indicate statistically significant support (*p* = 0.05) for a topology, and as such we also tested the significance threshold of 2.7 estimated by Ota et al. (2000)^91^, as well as a correction for multiple testing of 4.5^92^. These more stringent tests left very few partitions, which were not indecisive for the REA and WEA15 datasets but showed a clear signal of decaying support for Ctenophora sister and increasing support for Porifera sister from L1–L4 for the WEA17 dataset, consistent with the main analyses at lower thresholds (Supplementary Fig. 33). For recoded analyses amino acids were recoded according to SR4 coding^31^, and analysed in IQ-tree as though they were nucleotide data. Four-state recoding was chosen because IQ-tree does not permit the typically applied six-state recoding that has previously been applied in analyses of animal phylogeny. SR4 recoding has been used in many phylogenomic analyses (e.g. ^93^) and can also be taken to provide an independent line of evidence to analyses performed elsewhere with 6-state recodings. RT2 models incorporating site heterogeneity with recoding were built by summing the frequencies for the amino acids in each bin for each category^94,95^ (see also: https://groups.google.com/g/iqtree/c/j884eSJiugY/m/pH49d2S-CwAJ).

### Reporting summary

Further information on research design is available in the Nature Research Reporting Summary linked to this article.

## Supplementary information

Supplementary Information

Reporting Summary

## Data Availability

Alignments, best-fit model and partition files, new SR4-recoded model files and partition-specific log-likelihood value files have been uploaded to FigShare (10.6084/m9.figshare.12746972.v1).
