# Author Correction: Evidence for sponges as sister to all other animals from partitioned phylogenomics with mixture models and recoding

**DOI:** 10.1038/s41467-022-33707-w

**Published:** 2022-11-04

**Authors:** Anthony K. Redmond, Aoife McLysaght

**Affiliations:** Smurfit Institute of Genetics, Trinity College Dublin, Dublin, Ireland

**Keywords:** Phylogeny, Molecular evolution, Phylogenetics

Correction to: *Nature Communications* 10.1038/s41467-021-22074-7 published online 19 March 2021

The original version of this article reported highly unusual topologies when applying ‘R20’ 20% relaxed hierarchical clustering partition-finding to the ‘BEA’, ‘LEAN’ and ‘LEAP’ test datasets. This derived from a bug with IQ-tree version 1.5.4-omp (discovery of which was prompted by Nathan V. Whelan and Kenneth M. Halanych). The manuscript (including figshare repository data files) has now been edited with relevant reanalyses using an updated IQ-tree version (1.6.12), resulting in the following amendments to the text and figures:

- Figure 3 and its legend, which highlighted the unusual nature of these topologies, have now been removed. The previous Figs. 4–6 have now been changed to Figs. 3–5, respectively. All references to these figures in the main and Supplementary text have been updated to reflect this.
- Supplementary Table 2 and Supplementary Figs. 11–16 that presented data from our 20% relaxed hierarchical clustering partition-finding analyses have been updated with the results recovered when analyses were performed with IQ-tree v. 1.6.12.
- Supplementary Figs. 17–19 (and associated legends) that presented additional analyses to further investigate the unusual topologies have been removed. The previous Supplementary Figs. 20–36 have now been renamed to Supplementary Figs. 17–33, respectively. All references to these figures in the main and Supplementary text have been updated to reflect this.
- The latter half of the Results subsection ‘Standard partitioning approaches may be problematic in phylogenomics’ has been updated from:
  “Strikingly, for all three datasets re-analyses at L1 produced tree topologies that appear to be grossly incorrect. These analyses did not recover many expected relationships between species, such as the monophyly of eukaryotes or fungi in the BEA dataset, and the monophyly of animals, fungi, or any of the clades whose relationships are under investigation in the LEAP and LEAN datasets (Fig. 3a, Supplementary Figs. 11–13). These findings suggest that all of these analyses may have been affected by lumping errors (i.e. too many genes that are not best modelled under a single site-homogeneous model are clustered together)^61^. However, analyses of the same partitioning schemes but employing better-fitting site-heterogeneous models at L2-L4, RL1, and RL2 resolved this issue for all datasets (Fig. 3a, Supplementary Figs. 11–16), but were otherwise very similar to the results obtained when partitioning by gene alone, offering no additional improvement in LBA resilience.
  At odds with this however, unpartitioned analyses did not produce this apparently erroneous topology, and upon further examination we found that very few genes were clustered into larger partitions using 20% relaxed hierarchical clustering (Supplementary Table 2). Hence, we performed additional clustering experiments, based on the most frequently best-fitting L1 model (LG+G) only (for efficiency) (Supplementary Table 2). We found that the same issues were recovered with the L1 topologies even when more stringent clustering was applied (Fig. 3b; Supplementary Figs. 17–19). Further, when these partitioning schemes were analysed with the most frequently best-fitting L2 model (UL3+G) instead, the gross topological errors were no longer observed, and the results are consistent with those at L2 partitioning by gene and at 20% relaxed hierarchical clustering (Fig 3b; Supplementary Figs. 17–19).
  In all, these results imply that partitioning (at least when using genes as the basic unit) may be a problematic strategy in phylogenomics, hindering the ability of site-heterogeneous models to resist LBA through overparameterization, and risking lumping errors through underparameterization when genes are erroneously clustered into larger partitions under site-homogeneous models.”
  to now read:
  “At all analysis levels this produced results similar to those obtained when partitioning by gene alone, offering no consistent improvement in LBA resilience, and proved less effective at suppressing LBA than unpartitioned site-heterogeneous analyses (Supplementary Figs. 11–16).
  In all, these results imply that partitioning (at least when using genes as the basic unit) may be a problematic strategy in phylogenomics, hindering the ability of site-heterogeneous models to resist LBA through overparameterization, without shielding fully against lumping errors through underparameterization when genes are erroneously clustered into larger partitions under site-homogeneous models^61^.”
- The first two sentences of the Methods subsection ‘Model fitting and phylogenomics’ have been modified from:
  “All model testing and phylogenomic analyses were performed in IQ-tree (v. 1.5.4-omp)^82–84^. Best-fit models were chosen according to the commonly applied Bayesian Information Criterion (BIC) in ModelFinder^85^ (as packaged in IQ-tree v. 1.5.4-omp) (-m TEST), as this has higher specificity and so should be more conservative when considering complex models than the other commonly applied method, the Akaike information criterion (AIC)^85^.”
  to now read:
  “All model testing and phylogenomic analyses were performed in IQ-tree (v. 1.5.4-omp, except for hierarchical clustering partition-finding analyses of test datasets and subsequent reanalyses with site-heterogeneous models and/or recoding which were performed with v. 1.6.12)^82–84^. Best-fit models were chosen according to the commonly applied Bayesian Information Criterion (BIC) in ModelFinder^85^ (as packaged in IQ-tree) (-m TEST), as this has higher specificity and so should be more conservative when considering complex models than the other commonly applied method, the Akaike information criterion (AIC)^85^.”.
- The following sentences were removed from the Methods subsection ‘Model fitting and phylogenomics’:
  “Additional relaxed clustering analyses were also performed at 25%, 50%, and 75%, as well as a non-relaxed, full clustering analysis. These additional clustering analyses were performed with the model fixed as LG+G (the most frequently best fitting model at L1) for efficiency and were also reanalysed with the model fixed as UL3+G (the most frequently best fitting model at L2).”
- The following sentence has been added to the end of the Acknowledgements section:
  “We are thankful to Nathan V. Whelan and Kenneth M. Halanych for highlighting an error with partition-finding by hierarchical clustering in a previous version of this article.”.
- Reference 96 (Kapli, P. et al. Lack of support for Deuterostomia prompts reinterpretation of the first Bilateria. *bioRxiv* (2020) 10.1101/2020.07.01.182915), which was only referred to in the now removed original Fig. 3 legend, has been removed.
- The second sentence from the seventh paragraph of Supplementary Note1 has been truncated from:
  “Under- and/or over-partitioning, both of which can produce incorrect topologies^10,11^, may be unavoidable so long as genes are used as the minimal units upon which to base partitioning, the former seemingly evidenced in our R20 analyses of the BEA, LEAN and LEAP test datasets with site-homogeneous models (Fig. 3).”
  to instead read:
  “Under- and/or over-partitioning, both of which can produce incorrect topologies^10,11^, may be unavoidable so long as genes are used as the minimal units upon which to base partitioning.”

## Supplementary information

Updated Supplementary Information

