# Supplementary material for: Evidence for sponges as sister to all other animals from partitioned phylogenomics with mixture models and recoding
Source: Nat Commun. 2021 Mar 19;12:1783. doi: 10.1038/s41467-021-22074-7 (PMC7979703; doi:10.1038/s41467-021-22074-7)
Supplement: Supplementary file 1 — Supplementary Information [file 41467_2021_22074_MOESM1_ESM.pdf]

Evidence for sponges as sister to all other animals from partitioned Phylogenomics with mixture models and recoding

Anthony K. Redmond & Aoife McLysaght

Supplementary information

Supplementary Note 1: Consideration of the role of under- and over-fitting when using complex modelling strategies in partitioned phylogenomics to root animal phylogeny

An important consideration when applying complex models is the possibility of overfitting the data. Models that overfit the data may to result in a reduction in support for the correct topology due to increased variance, while on the other hand models that underfit the data may result in strong support for an incorrect topology due to increased bias[1]. The potential for positively misleading results (LBA) arguably makes the latter a more serious concern, and this is the scenario we, and others[2–4], infer for Ctenophora sister appearing when simplistic site-homogeneous models are used.

Our L1-L4 amino acid analyses never result in strong support for Porifera sister but show declining support for Ctenophora sister for the REA and WEA15 datasets (Fig. 4), which could at face value be argued to be caused by increased variance due to overfitting. However, multiple lines of evidence indicate that even if there is some overfitting, support for Porifera sister emerges independent to this.

We used the same model selection framework as is normally applied in partitioned phylogenomics, relying on the Bayesian information criterion (BIC) to select the models with the best relative fit to each partition at each analysis level. While the use of AIC and BIC for model selection has received some recent criticism[5], the potential for overfitting when they favour choosing, say UL3 or LG+C50 over LG or WAG, is not fundamentally different from when LG or WAG, which are almost always the best-fitting site-homogeneous models for animal partitioned datasets[3,6,7], are selected over simpler models like Dayhoff or Poisson.

In our analyses we found that increasingly more complex models are typically selected as best-fitting as they become available from analysis level L1-L4 (Fig. 4),

and that this roughly correlates with a shift in support, that is particularly apparent at the partition-specific level (Fig. 5), away from Ctenophora sister and towards Porifera sister. This continuous improvement in model-fit and the high prevalence of the most complex models as best-fitting, paired with the shift in support, strongly implies that more complex models might yet fit the data better and shift support further away from Ctenophora and towards Porifera as sister to other animals. This pattern is still readily apparent even if the L4 analysis is excluded and most genes/partitions are analysed while treating less well-fitting models as best (Fig. 5b), strongly rejecting a role for overfitting. The independent nature of our results from the T4 models and L4 analyses is particularly relevant as it has recently been asserted that the usually better-fitting T4 models may overfit process heterogeneity in the data. This is because standard models that ignore site-heterogeneity, like LG, may already incorporate information resulting from site-heterogeneity in the real data from which they are estimated[8]. It has thus been suggested that, by combining such models with C10-C60 models (which were developed to accommodate site heterogeneity), the combined efforts of these models may cause over-fitting of the site-heterogeneity in the data[8]. While this may theoretically suggest that use of T4 models is inappropriate, we contend that a similar argument might be made when accommodating rate-heterogeneity across sites, for example, with a gamma distribution, as at least some commonly applied standard site-homogeneous models (e.g. JTT) were also estimated without accounting for rate heterogeneity across sites. Despite this, combining these models with a gamma distribution of rates across sites is used almost universally for phylogenetic inference because of the improved results it offers. In line with this, our results, as well as those of some previous studies, strongly imply that T4 models behave as expected on both real and simulated data[9], providing improved resilience against long-branch attraction artefacts.

Unlike our L1-L4 analyses, our RL2 analyses appear to show major signs of overfitting. This is because although some loss of signal is expected when using amino acid recoding, the backbone animal relationships are poorly resolved for the REA and WEA17 datasets at RL2 compared to RL1 (which is also recoded but does not also use site-heterogeneous models). Further supporting a role for overfitting, the branching order of animal phyla is typically dataset specific as well as having low support (e.g. Placozoa as sister to other animals [UFBOOT=56%] and Ctenophora sister to Bilateria [UFBOOT=45%] are present only in REA; Supplementary Fig. 21).

In keeping with this RL2 analyses always favoured models pairing the simpler F81 with C10-C60, rather than GTR. This is roughly equivalent to T3 models always being favoured over T4 models in the L4 amino acid analyses, which is clearly not the case. Despite this apparent overfitting, strong support was obtained for Porifera sister at RL2 for the WEA15 (UBOOT=95%; Supplementary Fig. 22) and WEA17 (UFBOOT=99%; Supplementary Fig. 23) datasets, as well as there being some evidence for Cnidaria+Placozoa for WEA15 (UBOOT=90%; Supplementary Fig. 22). Taken together with the shift towards Porifera sister observed at the partition-specific level for the L1-L4 analyses, and assuming that neither overfitting nor recoding drive positively misleading results rather than simply increasing variance and eroding signal, this provides clear evidence for an increase in support for Porifera sister, rather than simple amelioration of support for Ctenophora sister as would be predicted by overfitting.

Importantly, support in the datasets analysed here is only substantially affected for those branches where site-homogeneous models are predicted to perform especially poorly (i.e. long branches such as Ctenophora, Xenacoelomorpha, or Microsporidia), whereas support remains unchanged for other non-suspect relationships (e.g. Ctenophora, Cnidaria, and Bilateria are all always monophyletic clades in our animal phylogeny analyses; Supplementary Figs. 17-19, and 21-23), implying that these branches are misplaced at L1 (at least) due to under-fitting of overly-simplistic models.

Despite these points, we suspect that some under- and/or over-fitting might occur in our analyses, but that this is primarily due to the partitioning approach itself. Under- and/or over-partitioning, both of which can produce incorrect topologies[10,11], may be unavoidable so long as genes are used as the minimal units upon which to base partitioning. Our comparisons between unpartitioned and partitioned analyses imply that partitioning can produce an overfitting effect (Supplementary Figs. 2, 4, and 6 versus Supplementary Figs. 8-10). Based on our test dataset analyses, and previous studies recovering support for Porifera sister using site-heterogeneous models, we predict that rather than acting as an explanation for the reducing support for Ctenophora sister from L1-L4, this effect instead hinders the shift in support from Ctenophora sister to Porifera sister from L1-L4. However, as this partition boundary induced overfitting pairs with possible underfitting of the heterogeneity within partitions, we suggest that the partitioned phylogenomics approach is affected by a

complex problem of combined over- and under-fitting. Thus, we advocate for the use of unpartitioned site-heterogeneous models, which also appears to perform better, as the best strategy to accommodate site profile variation in concatenated phylogenomics.

Finally, it is important to note that we do observe increased variance and/or reduced bias at the partition-specific level from L1-L4 (Fig. 5, Supplementary Fig. 26, 28 and 33). However, there is clear evidence for declining support for Ctenophora sister and increasing support for Porifera sister independent of this (Fig. 5, Supplementary Fig. 29).

In summary, the above points, together with other studies supporting Porifera sister rather than Ctenophora sister when more complex models are applied[2–4,12], clearly indicate that support for Ctenophora sister under simple models is an artefact of model underfitting, and that loss of support for Ctenophora sister in favour of Porifera sister under more complex models is not due to overfitting.

Supplementary References

1. Moran, R. J., Morgan, C. C. & O'Connell, M. J. A guide to phylogenetic reconstruction using heterogeneous models - A case study from the root of the placental mammal tree. *Computation* **3**, 177–196 (2015).
2. Pisani, D. *et al.* Genomic data do not support comb jellies as the sister group to all other animals. *Proc. Natl. Acad. Sci.* **112**, 201518127 (2015).
3. Feuda, R. *et al.* Improved Modeling of Compositional Heterogeneity Supports Sponges as Sister to All Other Animals. *Curr. Biol.* **27**, 3864-3870.e4 (2017).
4. Simion, P. *et al.* A Large and Consistent Phylogenomic Dataset Supports Sponges as the Sister Group to All Other Animals. *Curr. Biol.* **27**, 958–967 (2017).
5. Susko, E. & Roger, A. J. On the Use of Information Criteria for Model Selection in Phylogenetics. *Mol. Biol. Evol.* (2019) doi:10.1093/molbev/msz228.
6. Whelan, N. V, Kocot, K. M., Moroz, L. L. & Halanych, K. M. Error, signal, and the placement of Ctenophora sister to all other animals. *Proc. Natl. Acad. Sci. U. S. A.* **112**, 5773–8 (2015).
7. Whelan, N. V. *et al.* Ctenophore relationships and their placement as the sister group to all other animals. *Nat. Ecol. Evol.* **1**, 1737–1746 (2017).

8. Schrempf, D., Lartillot, N. & Szöllősi, G. Scalable empirical mixture models that account for across-site compositional heterogeneity. *bioRxiv* 794263 (2019) doi:10.1101/794263.
9. Wang, H. C., Minh, B. Q., Susko, E. & Roger, A. J. Modeling Site Heterogeneity with Posterior Mean Site Frequency Profiles Accelerates Accurate Phylogenomic Estimation. *Syst. Biol.* **67**, 216–235 (2018).
10. Seo, T. K. & Thorne, J. L. Information criteria for comparing partition schemes. *Syst. Biol.* **67**, 616–632 (2018).
11. Wang, H. C., Susko, E. & Roger, A. J. The Relative Importance of Modeling Site Pattern Heterogeneity Versus Partition-Wise Heterotachy in Phylogenomic Inference. *Syst. Biol.* **68**, 1003–1019 (2019).

Supplementary Tables

Supplementary Table 1. Summed log-likelihoods for the LBA and accepted topologies at each analysis level for test datasets.

**Dataset**	**BEA**		**LEAN**		**LEAP**	
**Hypothesis**	*LBA (Microsporidia sister to Eukaryotes)*	*Accepted (Microsporidia sister to/early branching Fungi)*	*LBA (Coelomata)*	*Accepted (Ecdysozoa)*	*LBA (Coelomata)*	*Accepted (Protostomia)*
**L1**	-729979.7334	-730286.5616	-724306.3185	-724350.3986	-635874.3866	-635965.7711
**L2**	-720640.4695	-720787.1399	-716171.4235	-716154.8867	-629431.6007	-629469.1412
**L3**	-719173.6748	-719235.4772	-714943.3637	-714919.0482	-628441.4362	-628457.4809
**L4**	-717989.8660	-717954.2605	-712100.5093	-712058.7123	-626336.3088	-626332.4867
**RL1**	-279306.4203	-279316.2031	-273972.5767	-273957.7447	-241950.6150	-242005.0063
**RL2**	-273908.8712	-273885.5077	-269823.6229	-269765.3223	-238804.7448	-238785.8082

Supplementary Table 2. Number and size of partitions after clustering analyses.

Dataset	Clustering	Genes in partition	Partitions of this size
**BEA**	*Gene*	1	133
	R20	1	23
		2	55
**LEAN**	*Gene*	1	146
	R20	16	1
		15	1
		14	1
		10	1
		9	1
		8	6
		5	2
		4	3
		3	2
		2	2
		1	2
**LEAP**	*Gene*	1	146
	R20	16	1
		14	1
		10	1
		8	3
		7	2
		5	1
		4	5
		3	9
		2	7
		1	2

Supplementary Figures

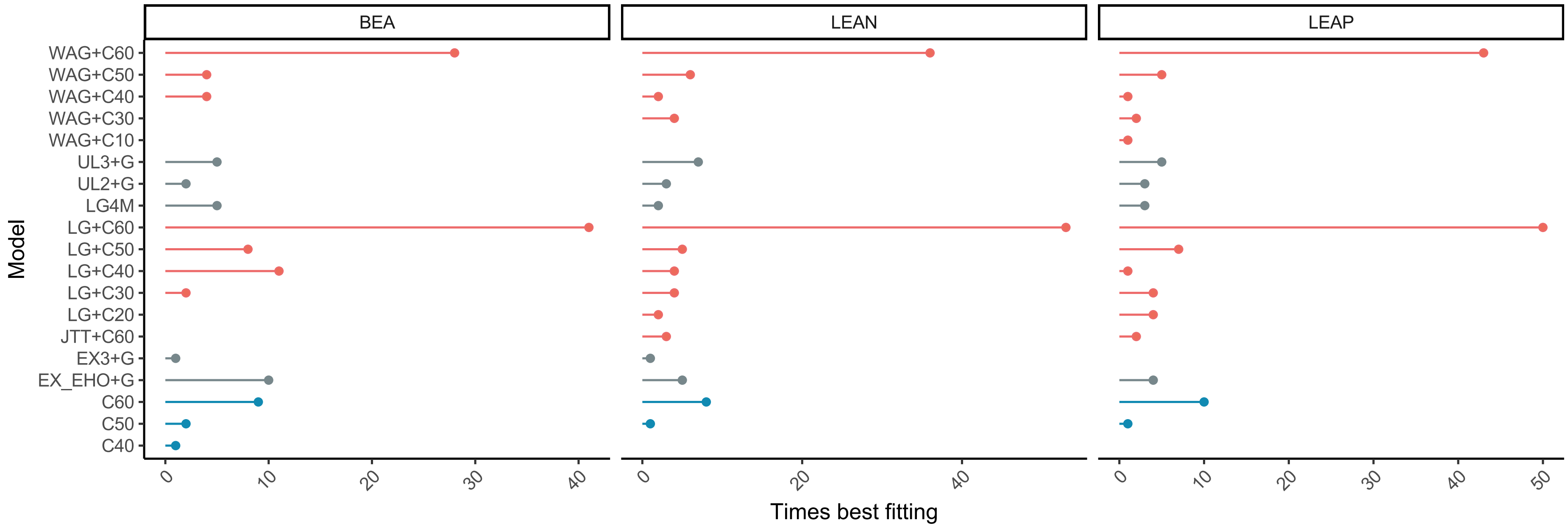

Supplementary Fig. 1. Number of partitions each model best-fits for the BEA and LEA(N/P) datasets. Colours represent model Tier (T1: light grey, T2: dark grey, T3: blue, T4: red).

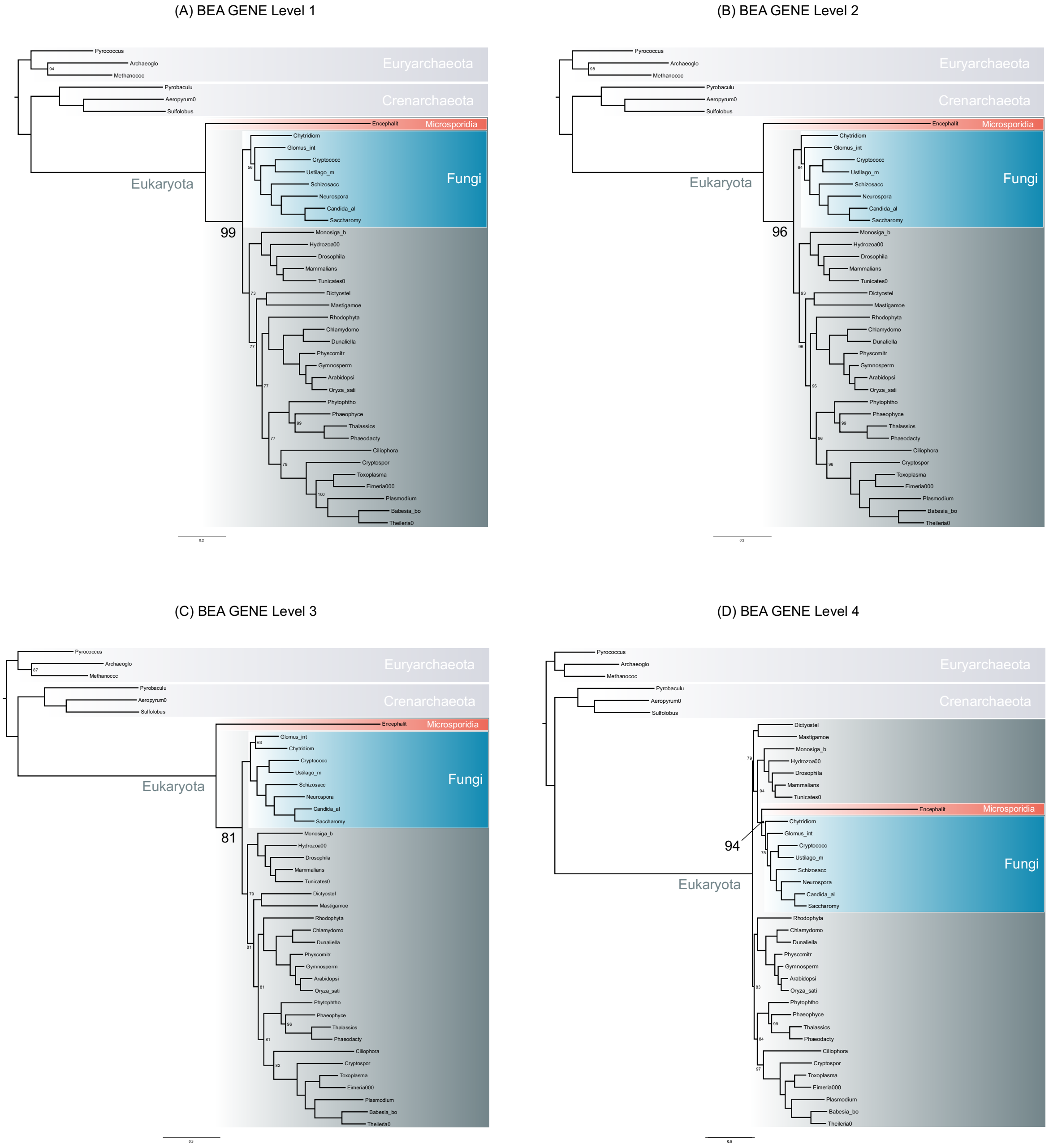

Supplementary Fig. 2. Full consensus trees with UFBOOT supports (not shown where maximal) from analyses of the BEA dataset partitioned by gene at analysis levels L1 (A), L2 (B), L3 (C), and L4 (D).

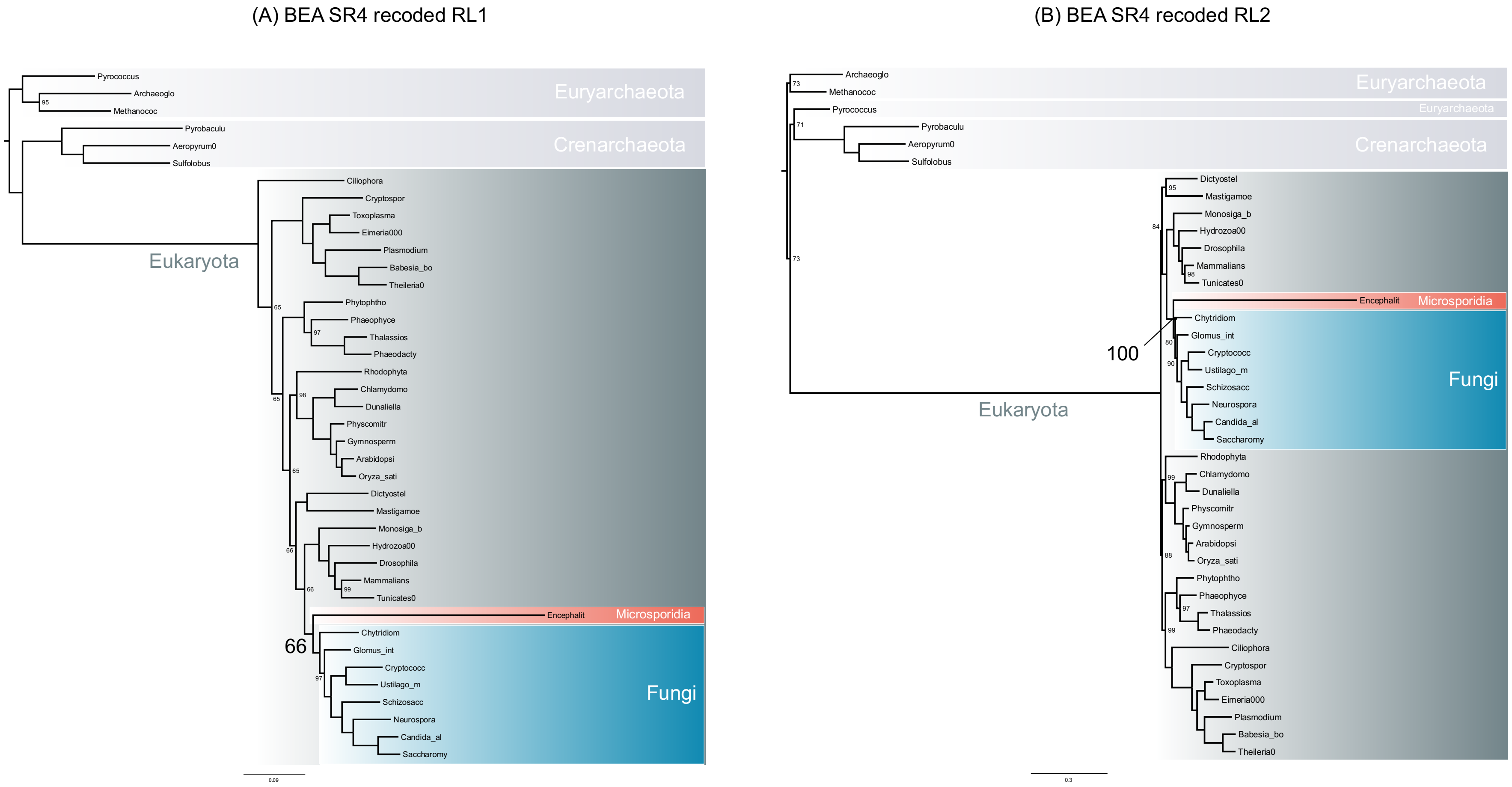

Supplementary Fig. 3. Full consensus trees with UFBOOT supports (not shown where maximal) from analyses of the SR4 recoded BEA dataset partitioned by gene at analysis levels RL1 (A) and RL2 (B).

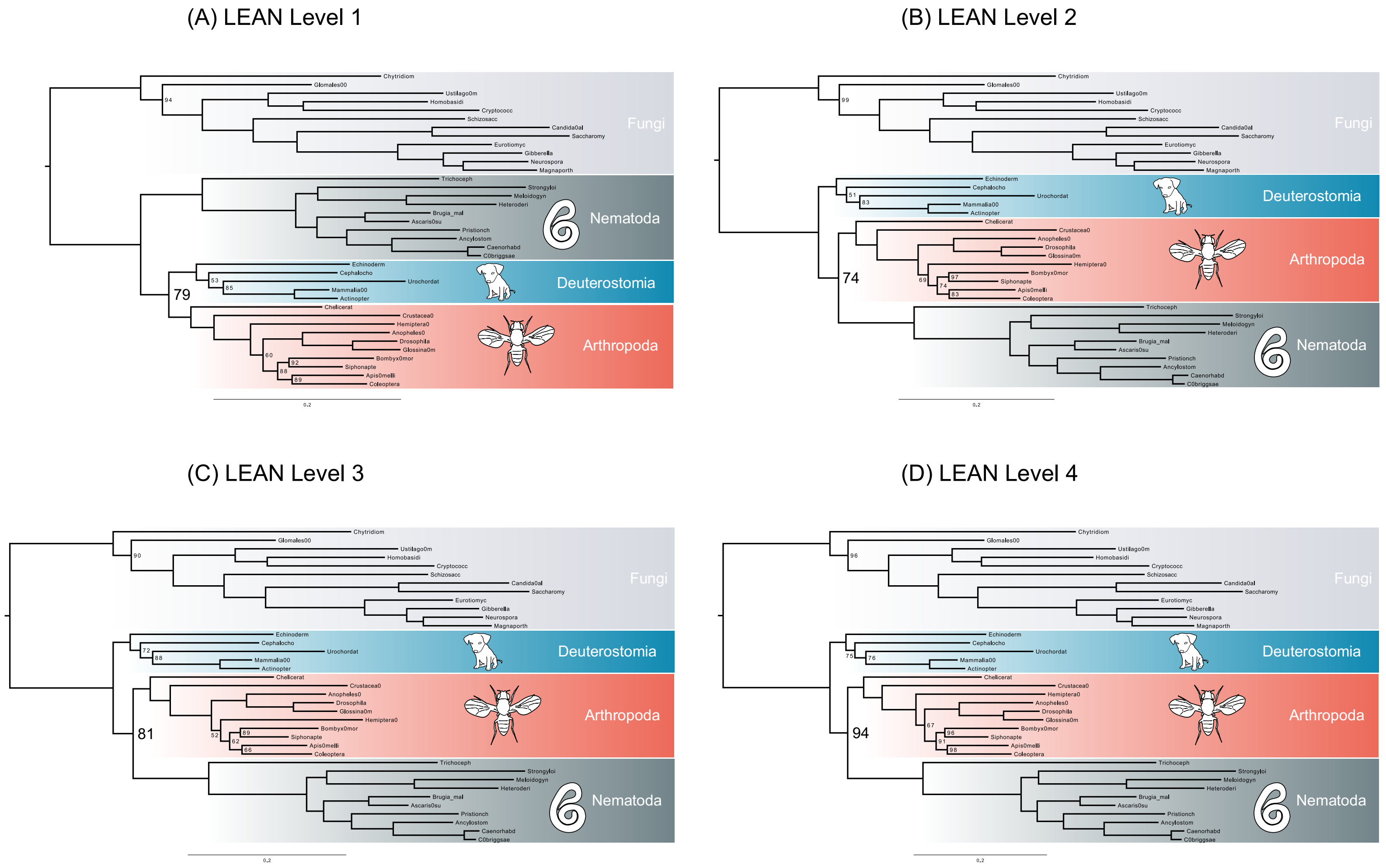

Supplementary Fig. 4. Full consensus trees with UFBOOT supports (not shown where maximal) from analyses of the LEAN dataset partitioned by gene at analysis levels L1 (A), L2 (B), L3 (C), and L4 (D).

(A) LEAN SR4 recoded RL1 (B) LEAN SR4 recoded RL2

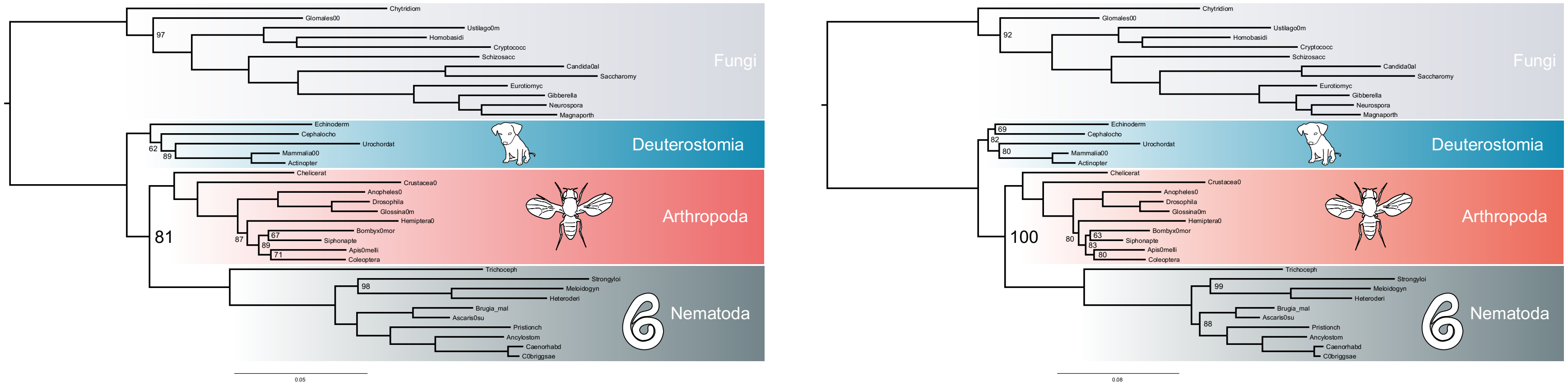

Supplementary Fig. 5. Full consensus trees with UFBOOT supports (not shown where maximal) from analyses of the SR4 recoded LEAN dataset partitioned by gene at analysis levels RL1 (A) and RL2 (B).

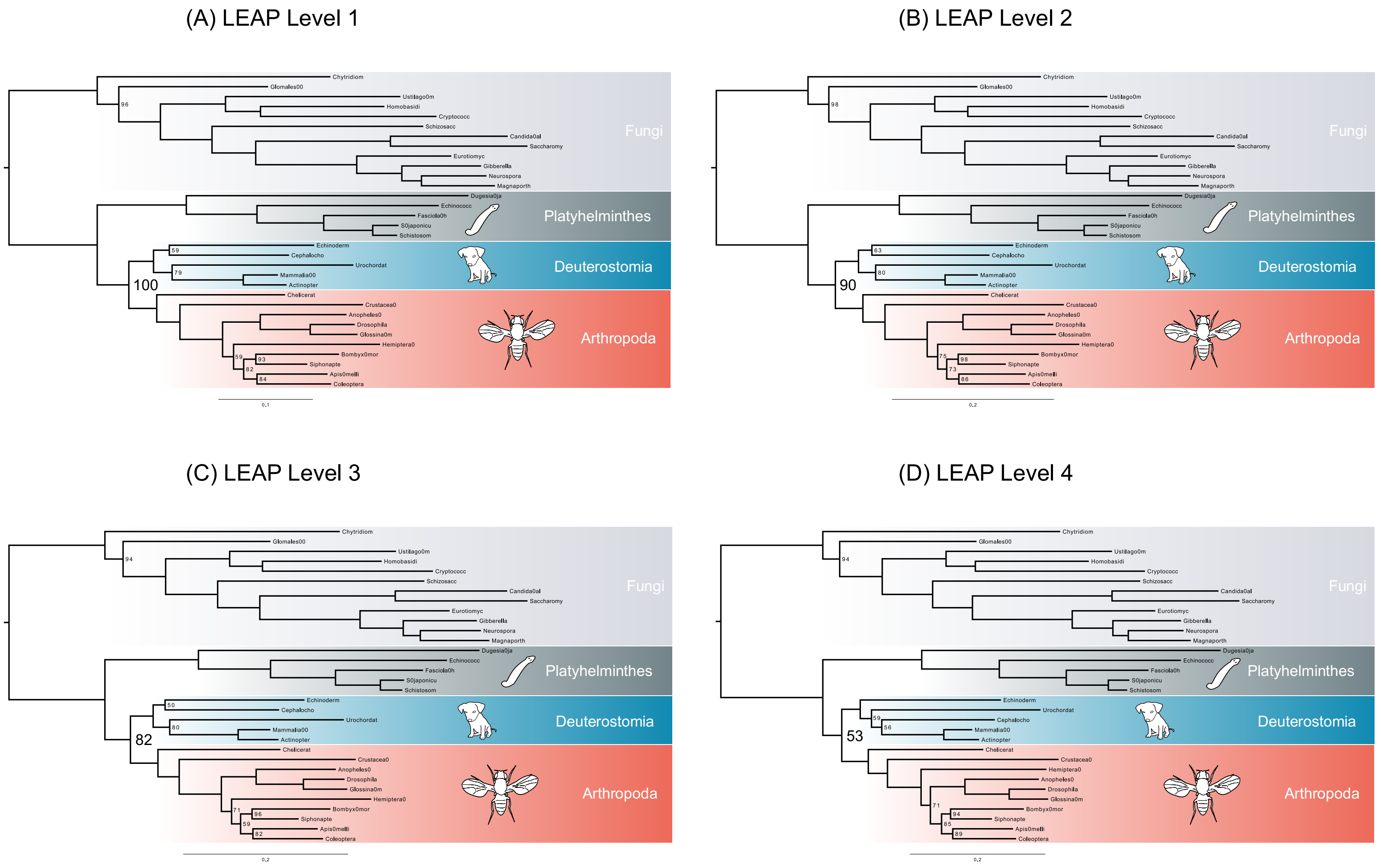

Supplementary Fig. 6. Full consensus trees with UFBOOT supports (not shown where maximal) from analyses of the LEAP dataset partitioned by gene at analysis levels L1 (A), L2 (B), L3 (C), and L4 (D).

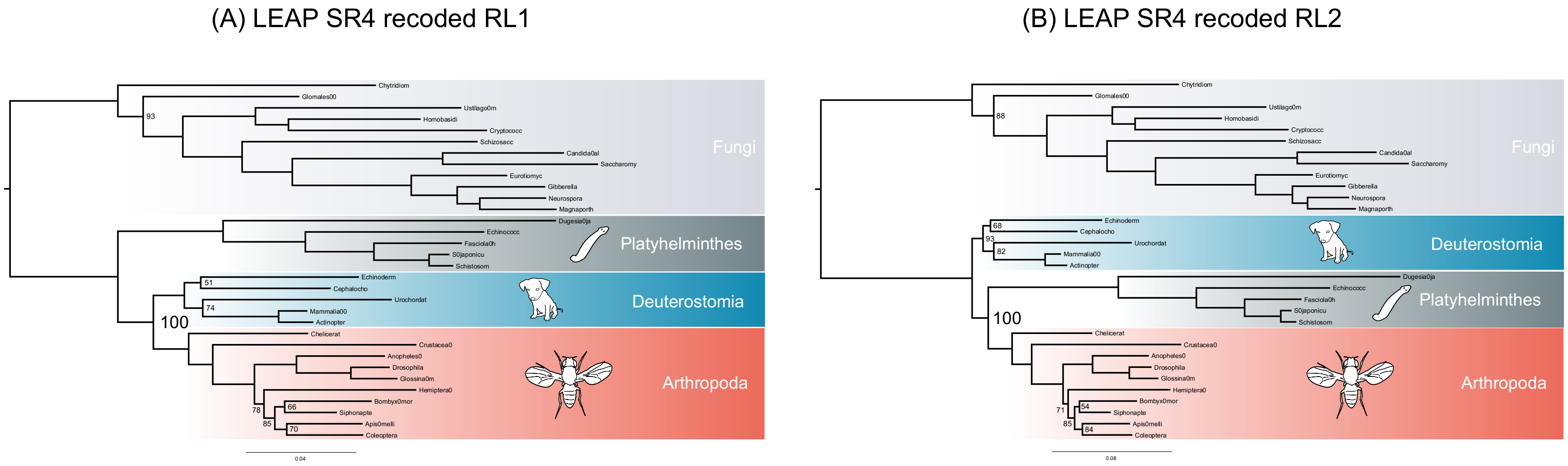

Supplementary Fig. 7. Full consensus trees with UFBOOT supports (not shown where maximal) from analyses of the SR4 recoded LEAP dataset partitioned by gene at analysis levels RL1 (A) and RL2 (B).

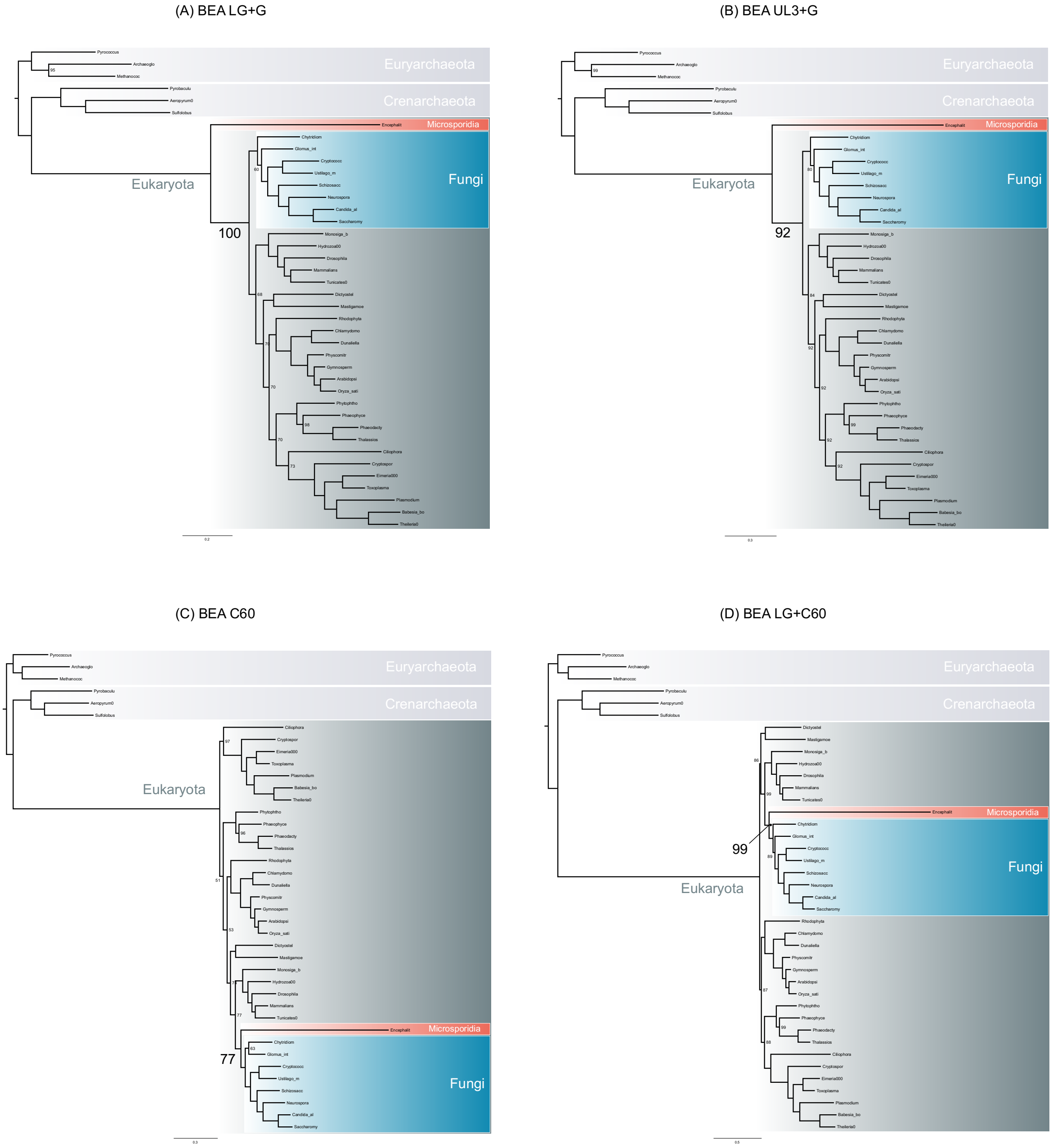

Supplementary Fig. 8. Full consensus trees with UFBOOT supports (not shown where maximal) from unpartitioned analyses of the BEA dataset using most frequently best-fitting models at L1 (A), L2 (B), L3 (C), and L4 (D).

(A) LEAN LG+G

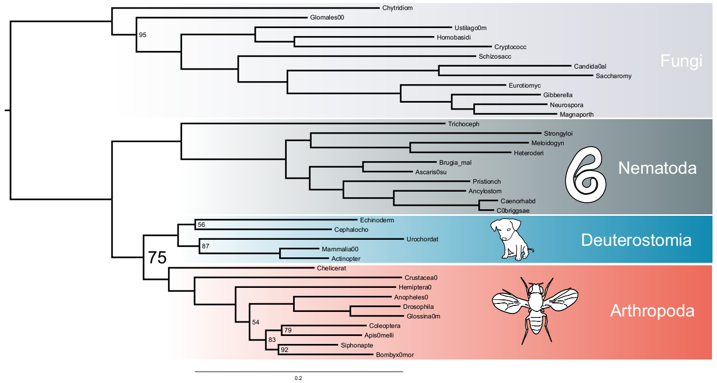

(B) LEAN UL3+G

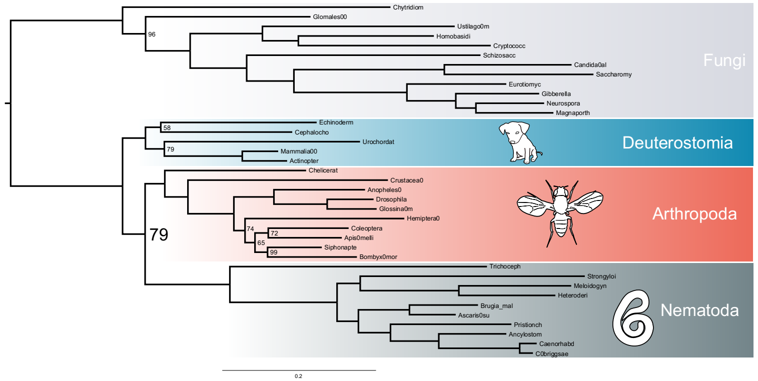

(C) LEAN C60

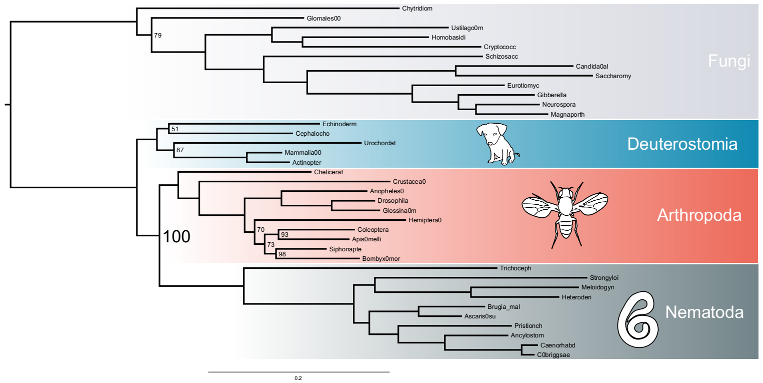

Supplementary Fig. 9. Full consensus trees with UFBOOT supports (not shown where maximal) from unpartitioned analyses of the LEAN dataset using most frequently best-fitting models at L1 (A), L2 (B), and L3 (C).

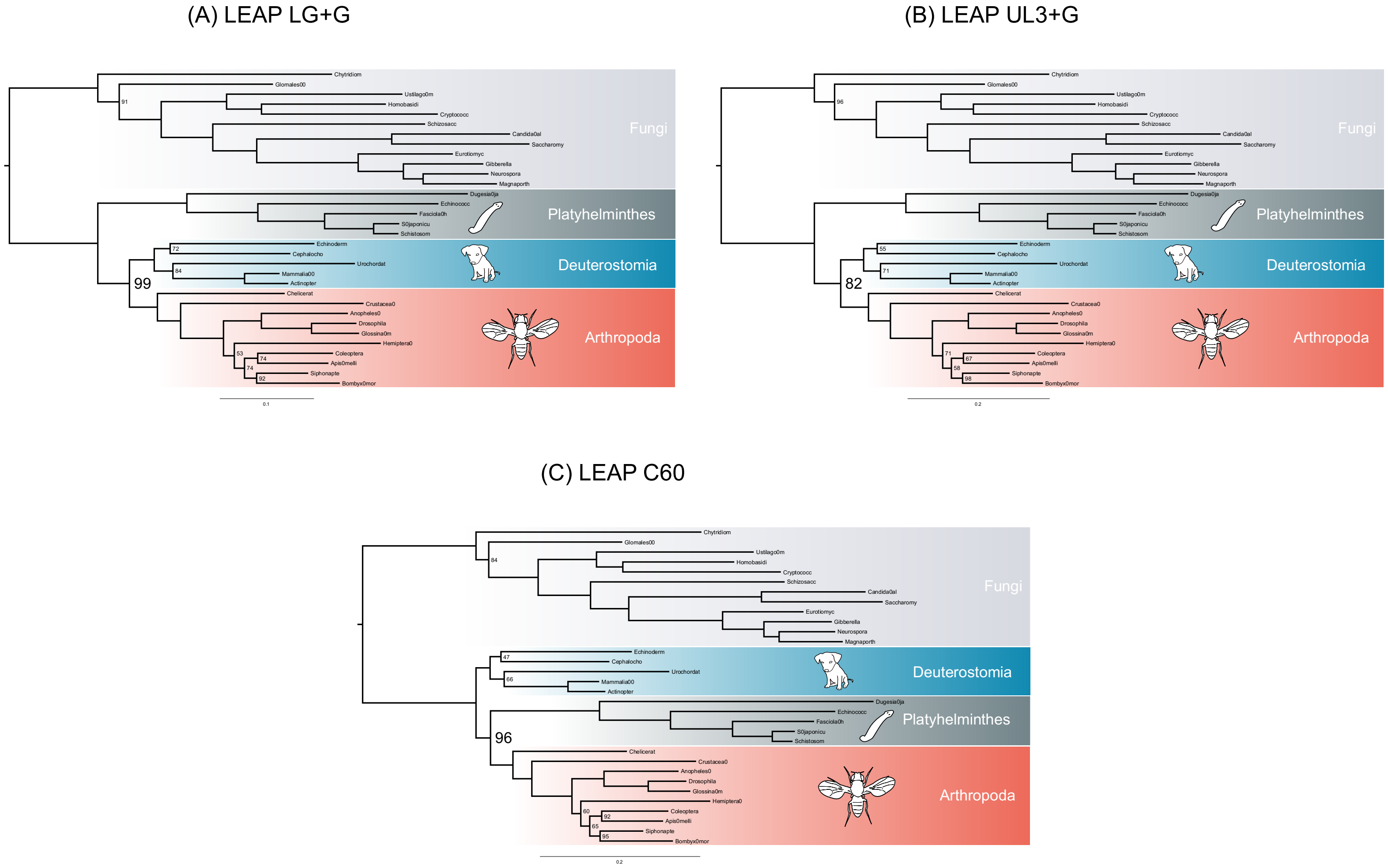

Supplementary Fig. 10. Full consensus trees with UFBOOT supports (not shown where maximal) from unpartitioned analyses of the LEAP dataset using most frequently best-fitting models at L1 (A), L2 (B), and L3 (C).

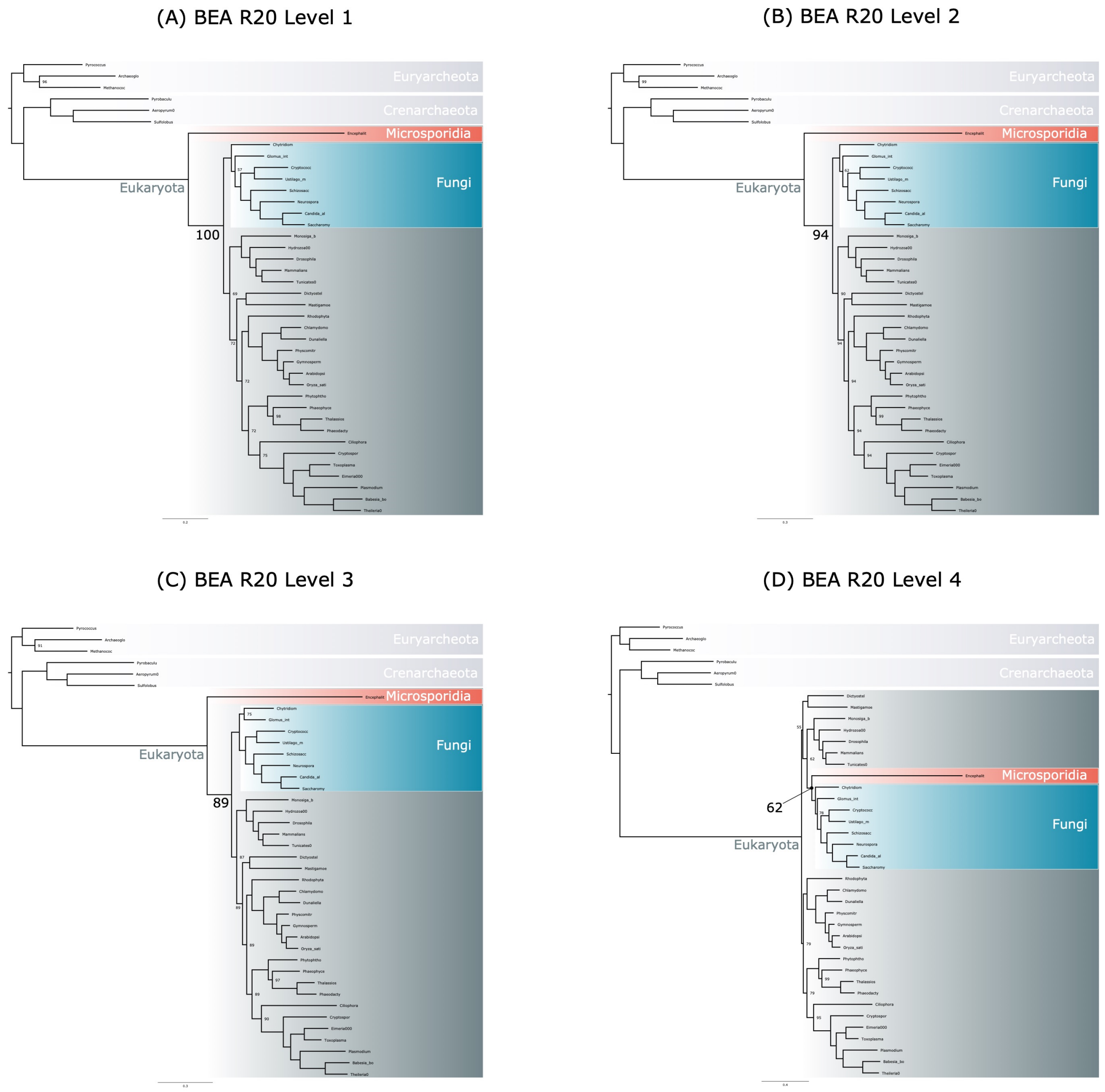

Supplementary Fig. 11. Full consensus trees with UFBOOT supports (not shown where maximal) from analyses of the BEA dataset with L1 R20 partitions at analysis levels L1 (A), L2 (B), L3 (C), and L4 (D).

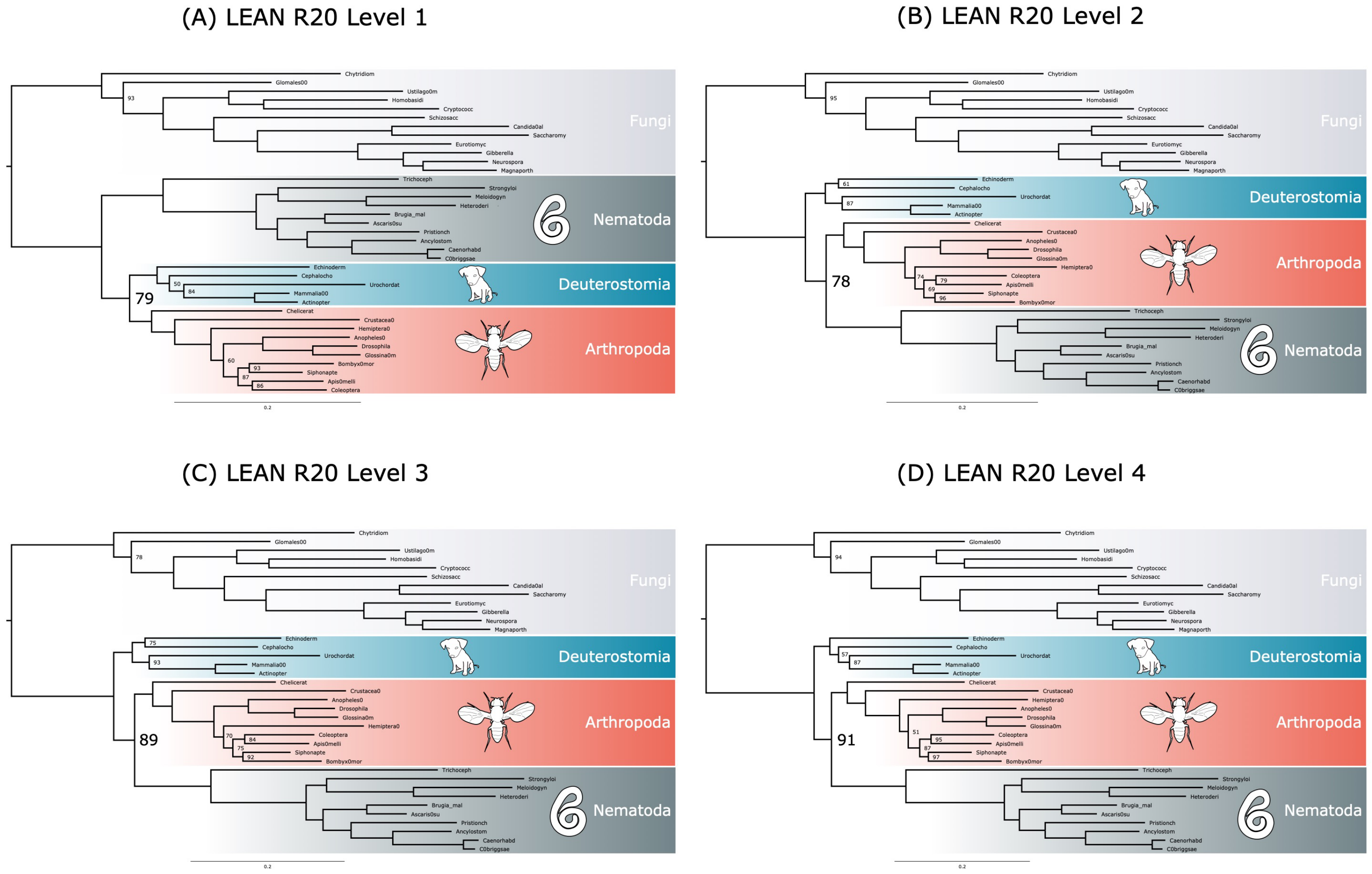

Supplementary Fig. 12. Full consensus trees with UFBOOT supports (not shown where maximal) from analyses of the LEAN dataset with L1 R20 partitions at analysis levels L1 (A), L2 (B), L3 (C), and L4 (D).

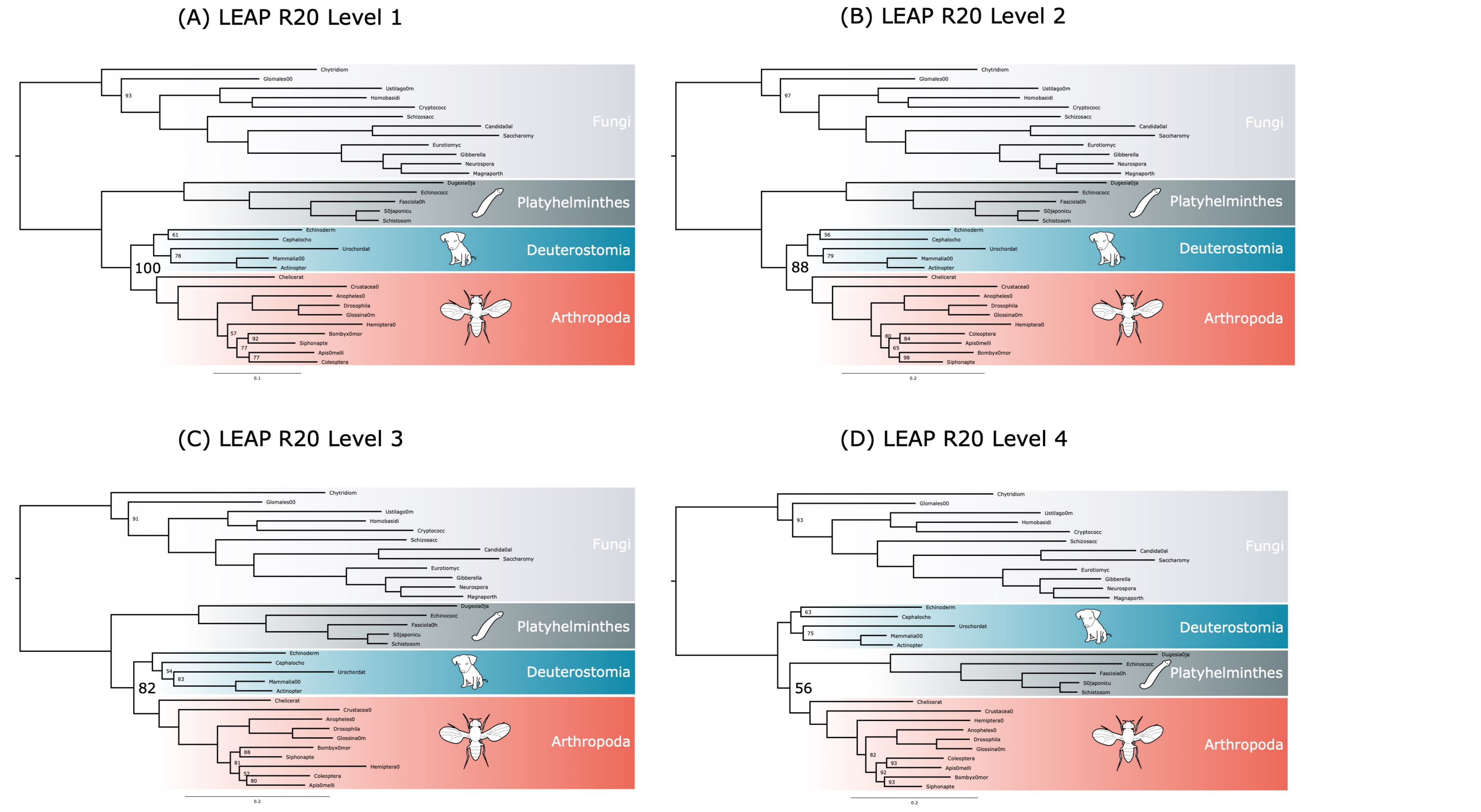

Supplementary Fig. 13. Full consensus trees with UFBOOT supports (not shown where maximal) from analyses of the LEAP dataset with L1 R20 partitions from analysis levels at analysis levels L1 (A), L2 (B), L3 (C), and L4 (D).

(A) BEA R20 SR4 recoded RL1 (B) BEA R20 SR4 recoded RL2

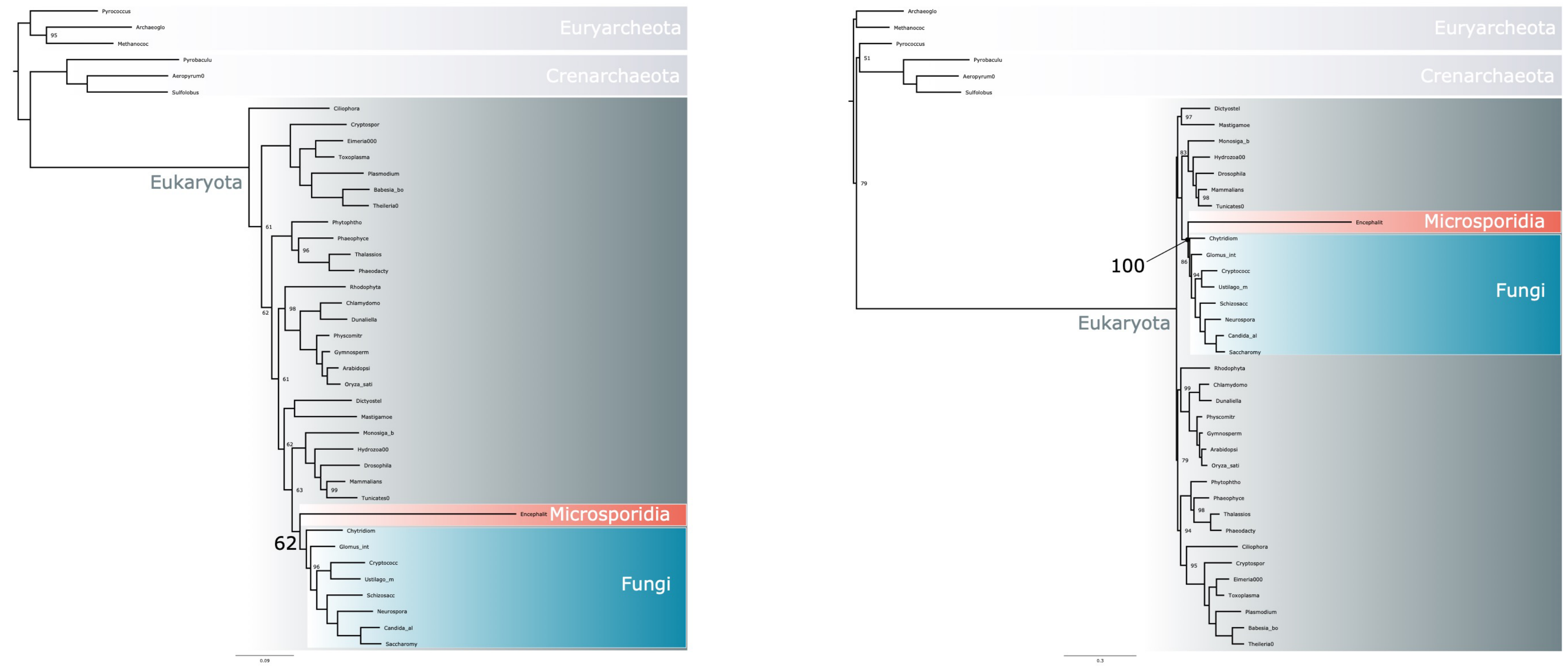

Supplementary Fig. 14. Full consensus trees with UFBOOT supports (not shown where maximal) from analyses of the BEA dataset with L1 R20 partition schemes at analysis levels RL1 (A) and RL2 (B).

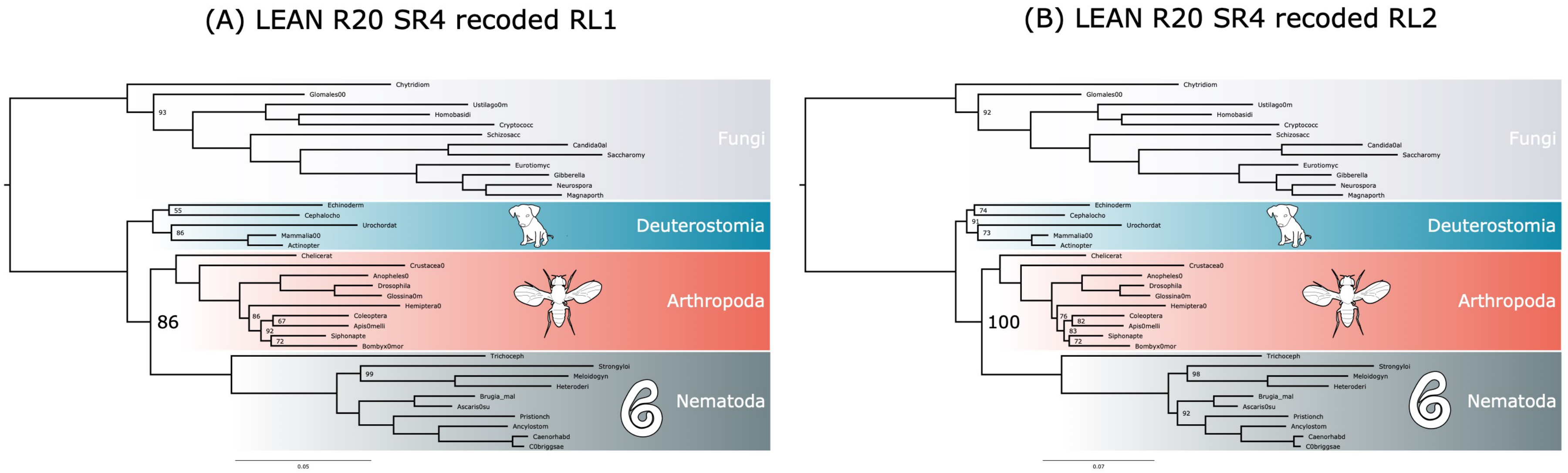

Supplementary Fig. 15. Full consensus trees with UFBOOT supports (not shown where maximal) from analyses of the LEAN dataset with L1 R20 partition schemes at analysis levels RL1 (A) and RL2 (B).

(A) LEAP R20 SR4 recoded RL1 (B) LEAP R20 SR4 recoded RL2

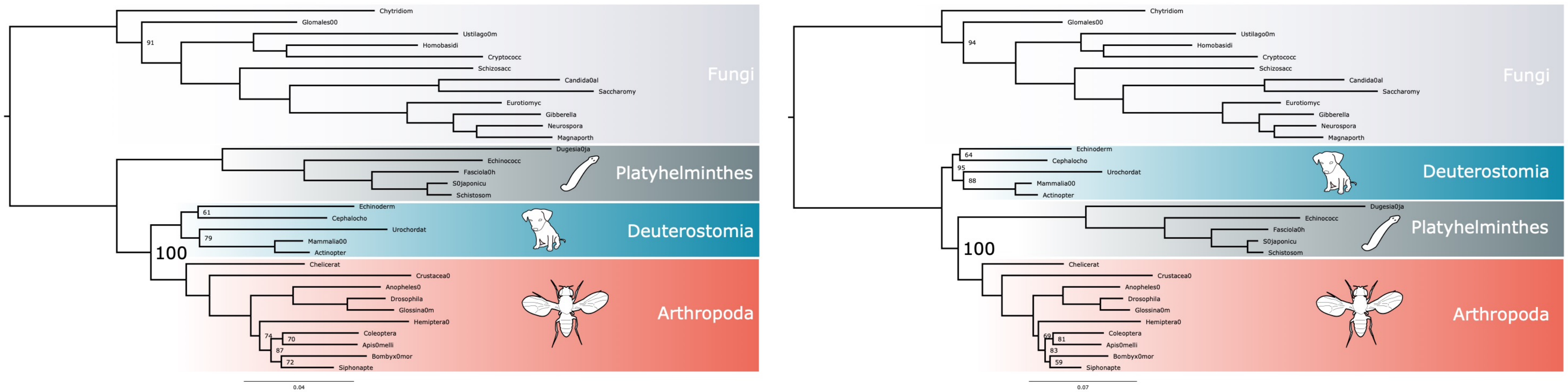

Supplementary Fig. 16. Full consensus trees with UFBOOT supports (not shown where maximal) from analyses of the LEAP dataset with L1 R20 partition schemes at analysis levels RL1 (A) and RL2 (B).

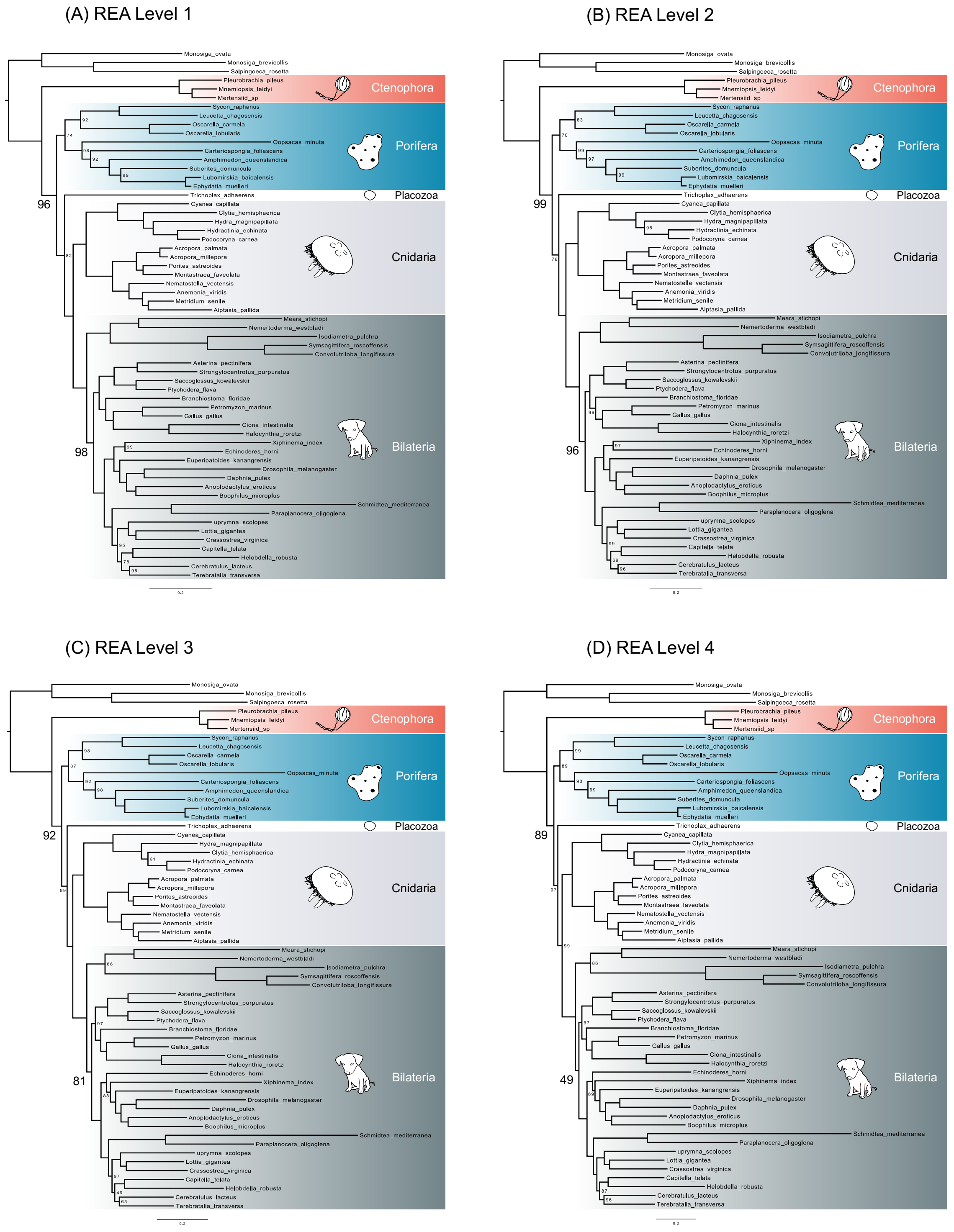

Supplementary Fig. 17. Full consensus trees with UFBOOT supports (not shown where maximal) from analyses of the REA dataset partitioned by gene at analysis levels L1 (A), L2 (B), L3 (C), and L4 (D).

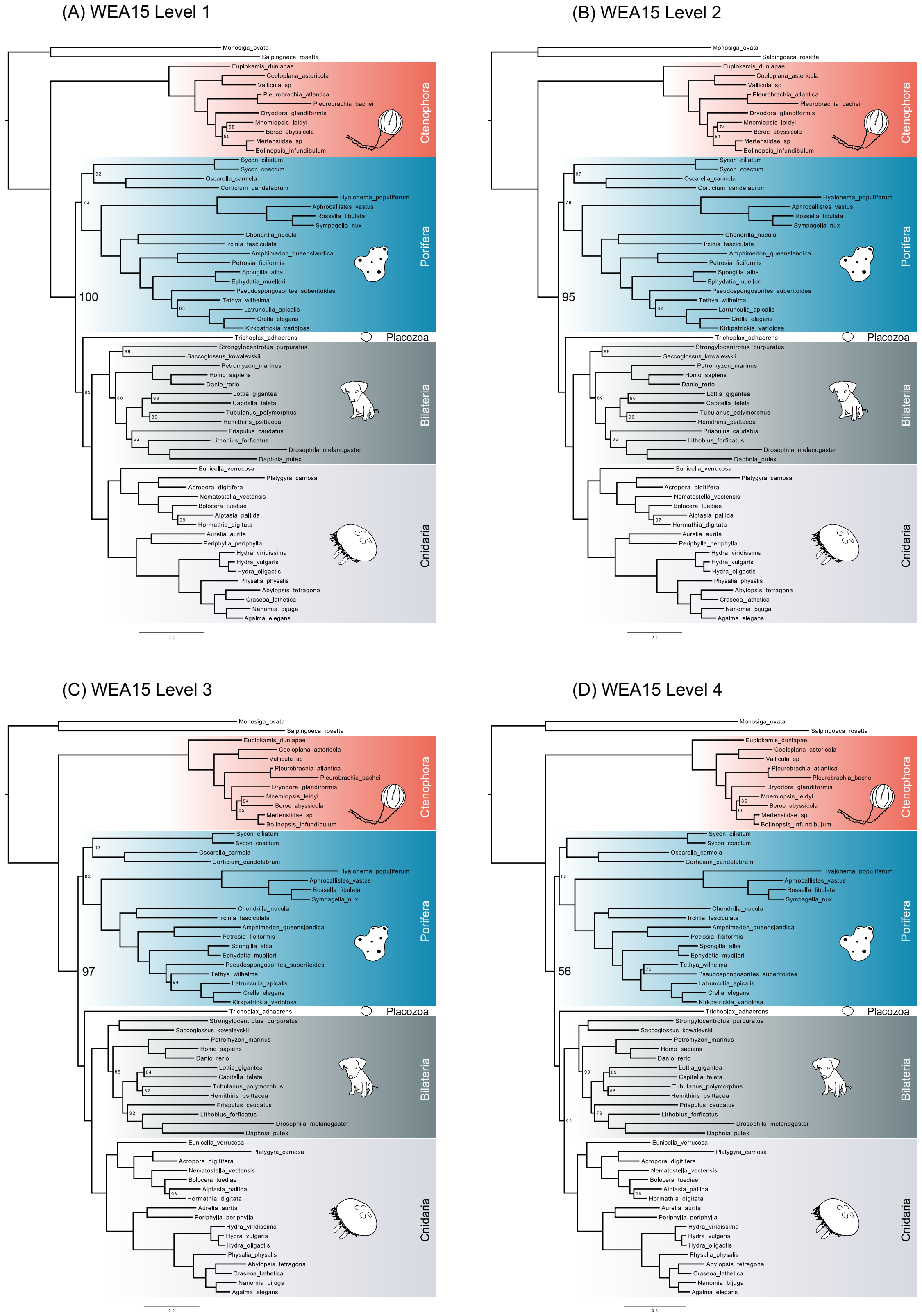

Supplementary Fig. 18. Full consensus trees with UFBOOT supports (not shown where maximal) from analyses of the WEA15 dataset partitioned by gene (which was also the best-fitting partitioning scheme from the original study) at analysis levels L1 (A), L2 (B), L3 (C), and L4 (D).

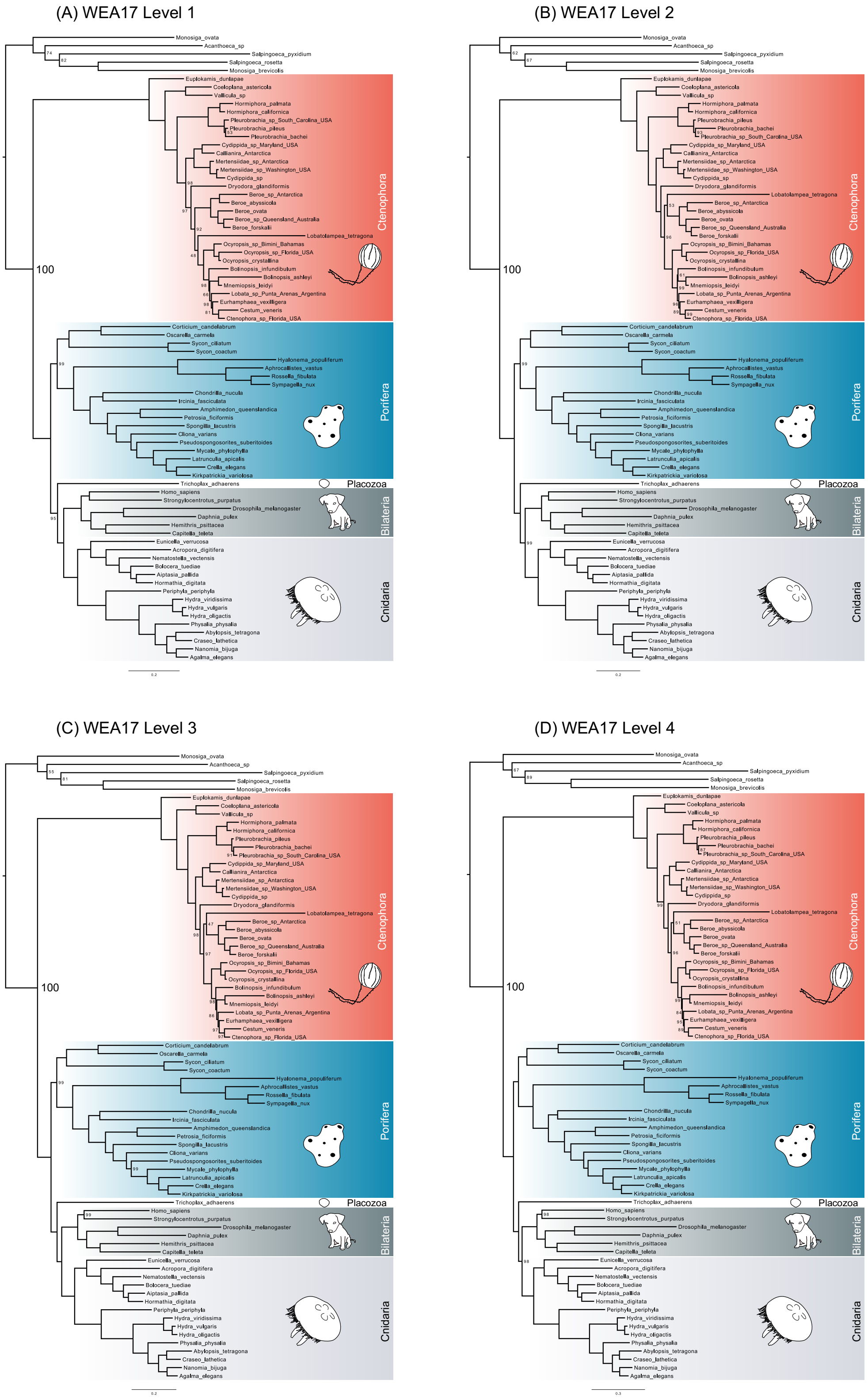

Supplementary Fig. 19. Full consensus trees with UFBOOT supports (not shown where maximal) from analyses of the WEA17 dataset partitioned according to the original study at analysis levels L1 (A), L2 (B), L3 (C), and L4 (D).

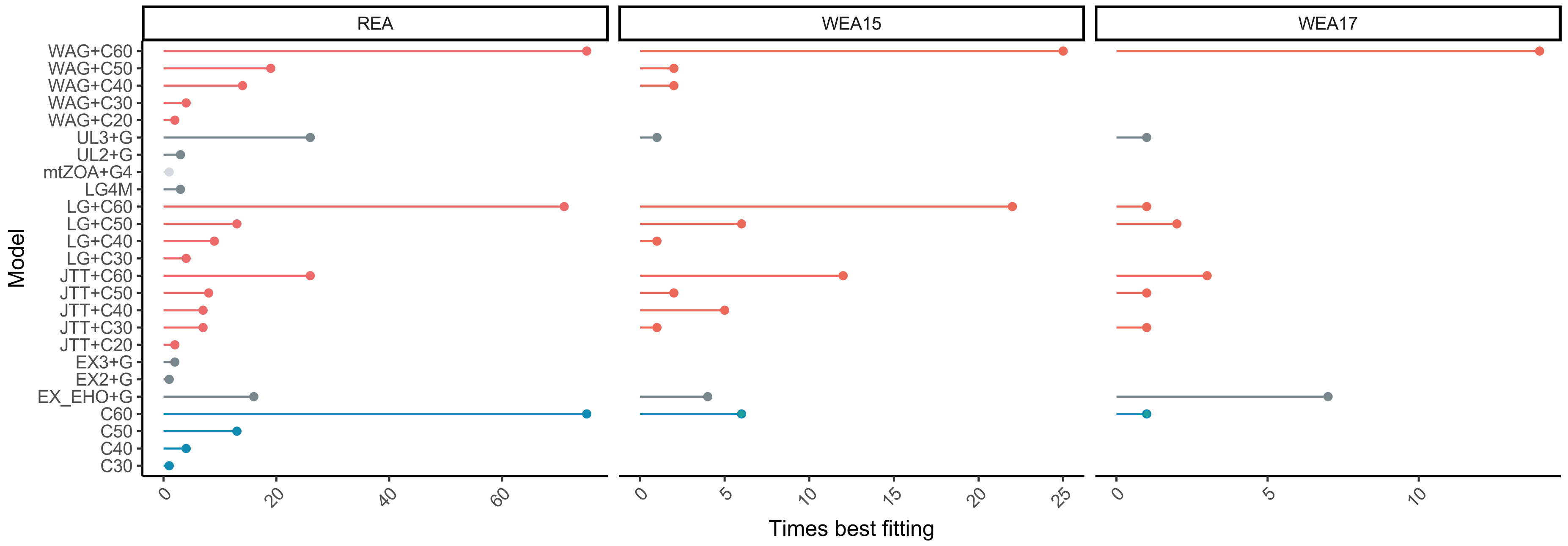

Supplementary Fig. 20. Number of partitions each model best-fits for the REA and WEA15, and WEA17 datasets. Colours represent model Tier (T1: light grey, T2: dark grey, T3: blue, T4: red).

(A) REA SR4 recoded RL1 (B) REA SR4 recoded RL2

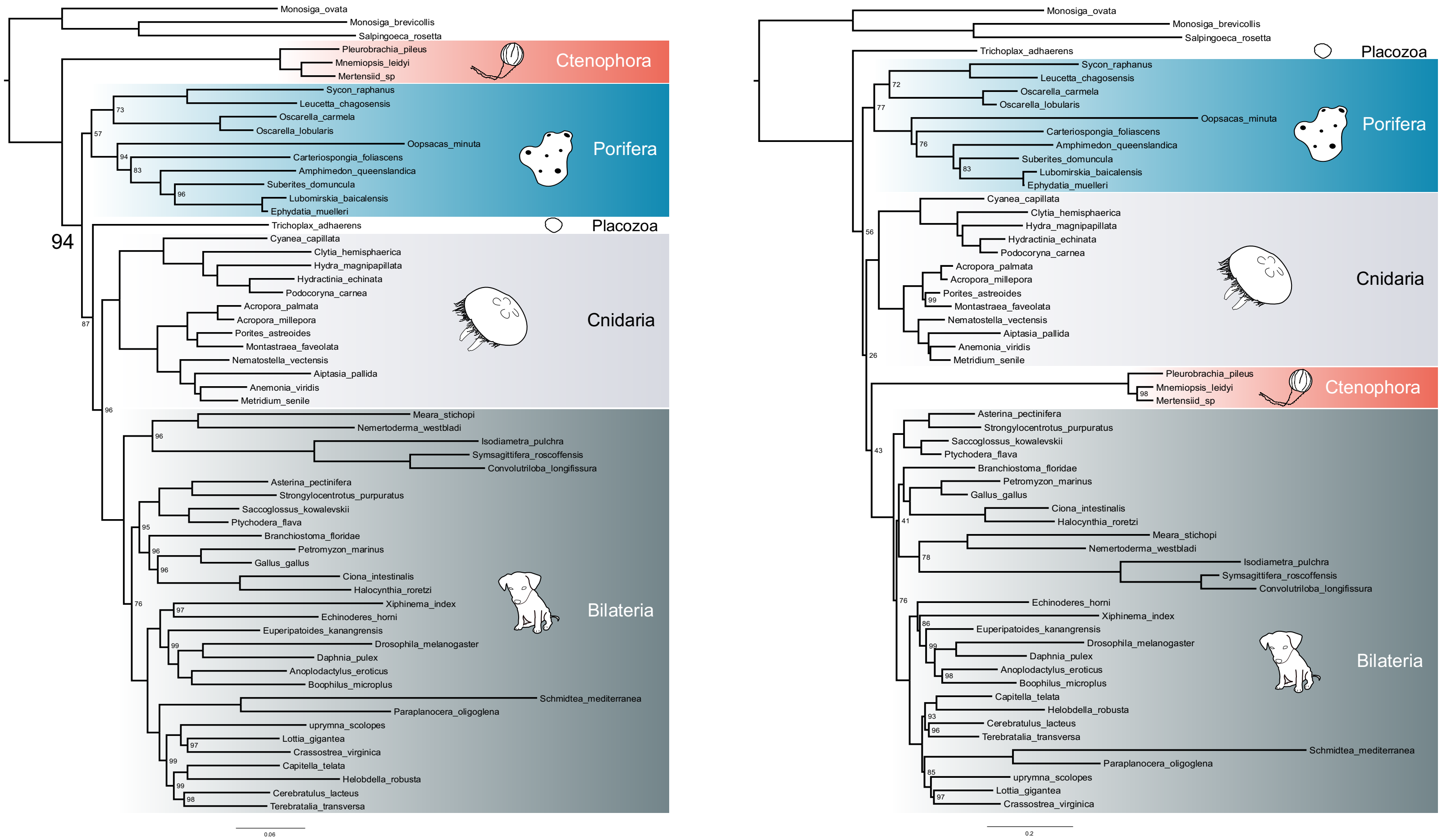

Supplementary Fig. 21. Full consensus trees with UFBOOT supports (not shown where maximal) from analyses of the SR4 recoded REA dataset partitioned by gene at analysis levels RL1 (A) and RL2 (B).

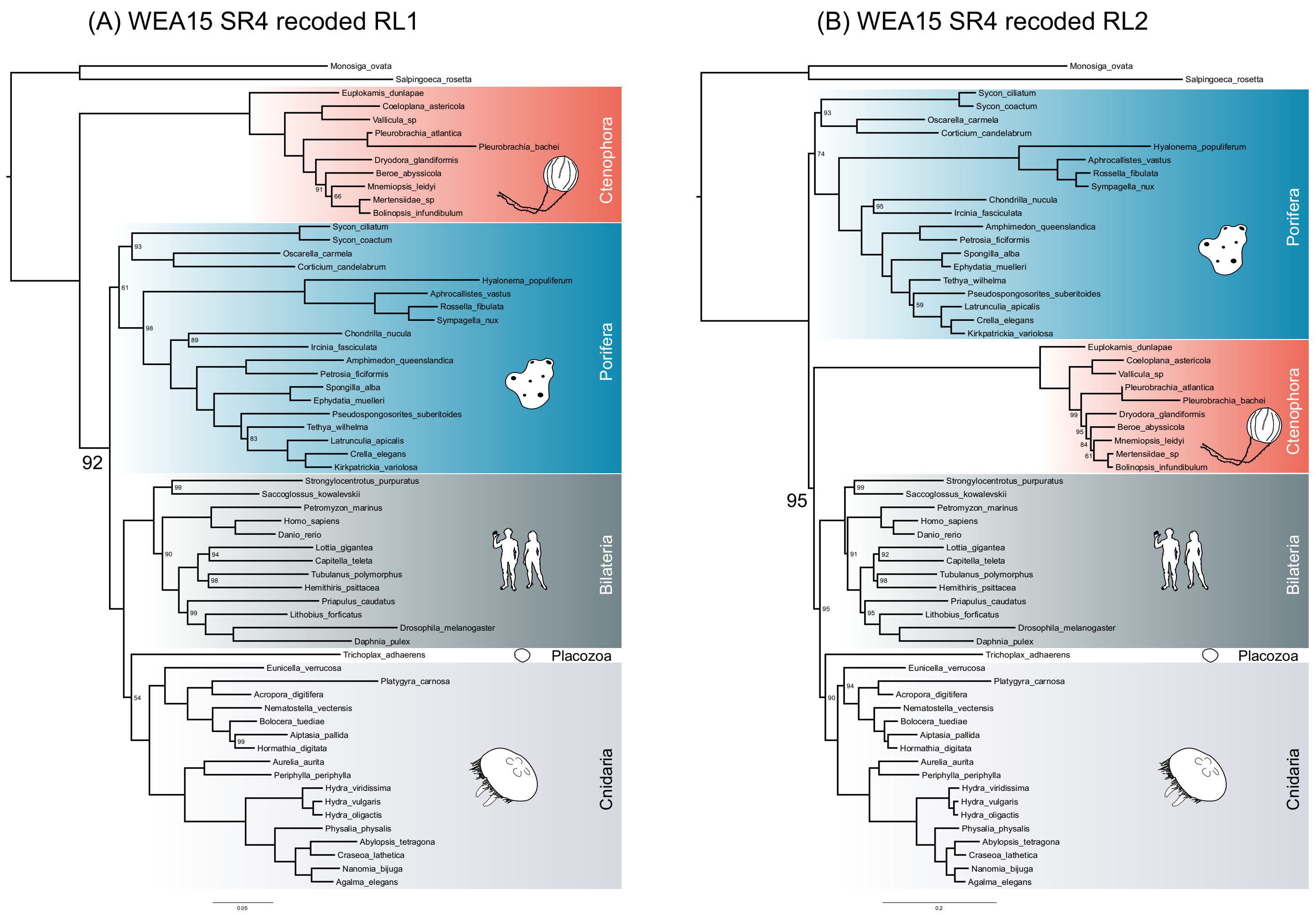

Supplementary Fig. 22. Full consensus trees with UFBOOT supports (not shown where maximal) from analyses of the SR4 recoded WEA15 dataset partitioned by gene (which was also the best-fitting partitioning scheme from the original study) at analysis levels RL1 (A) and RL2 (B).

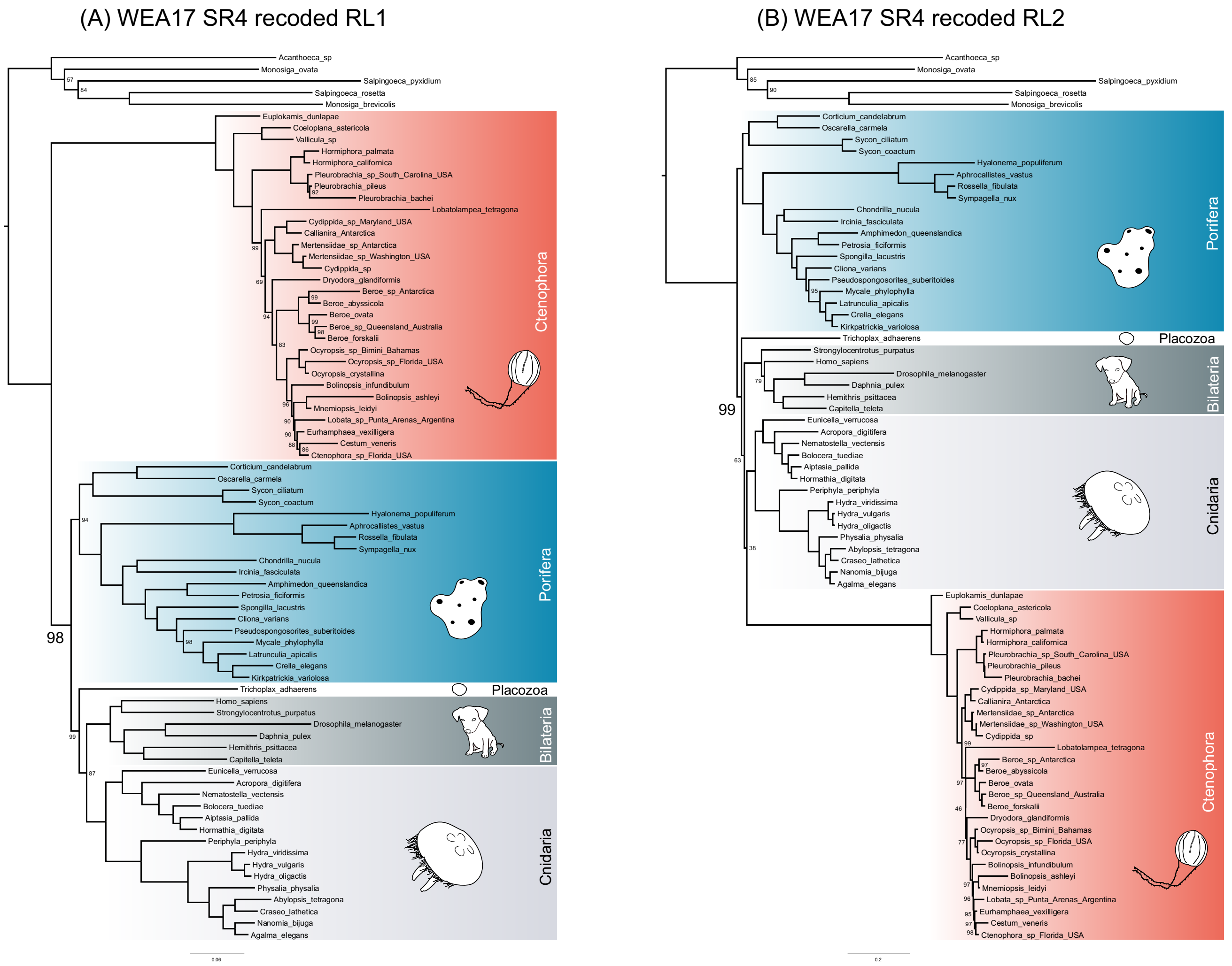

Supplementary Fig. 23. Full consensus trees with UFBOOT supports (not shown where maximal) from analyses of the SR4 recoded WEA17 dataset partitioned according to the original study at analysis levels RL1 (A) and RL2 (B).

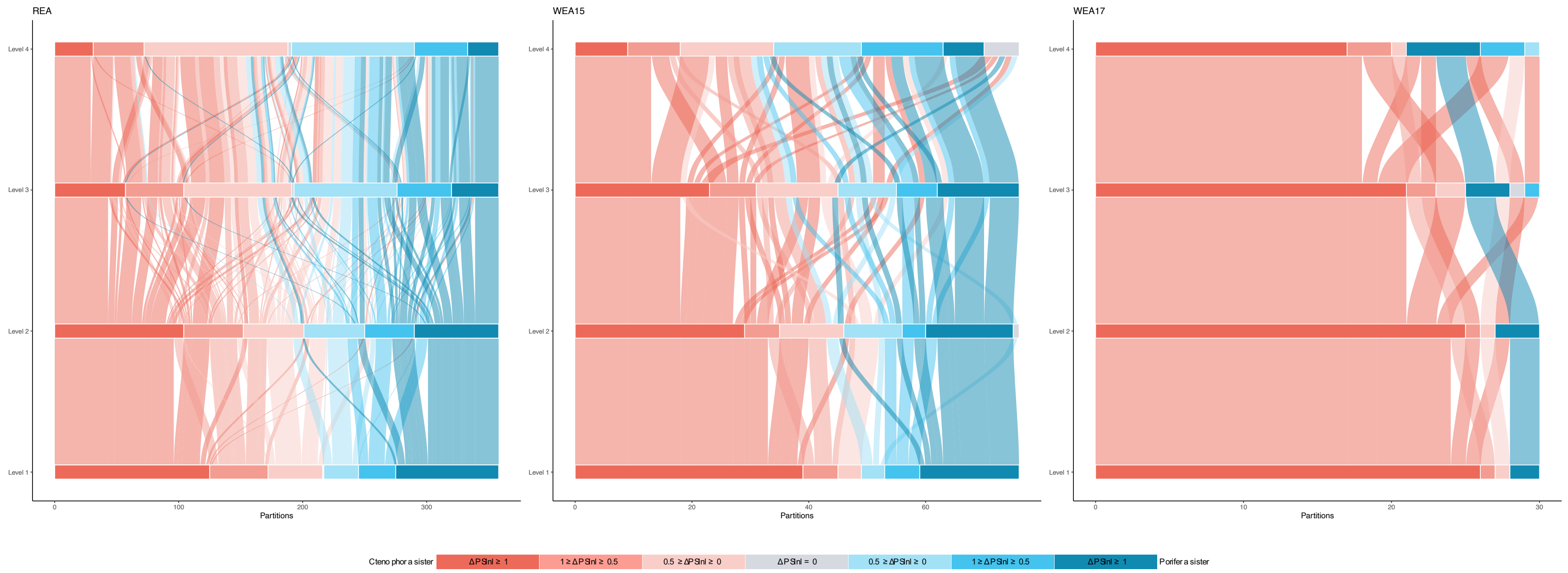

Supplementary Fig. 24. Alluvial plots showing the proportional flow of partitions between the defined ΔPSlnl hypotheses and support brackets from analysis levels L1 to L4 for the three animal phylogeny datasets. Horizontal bars plot the proportion of partitions with support values in each bracket at each analysis level.

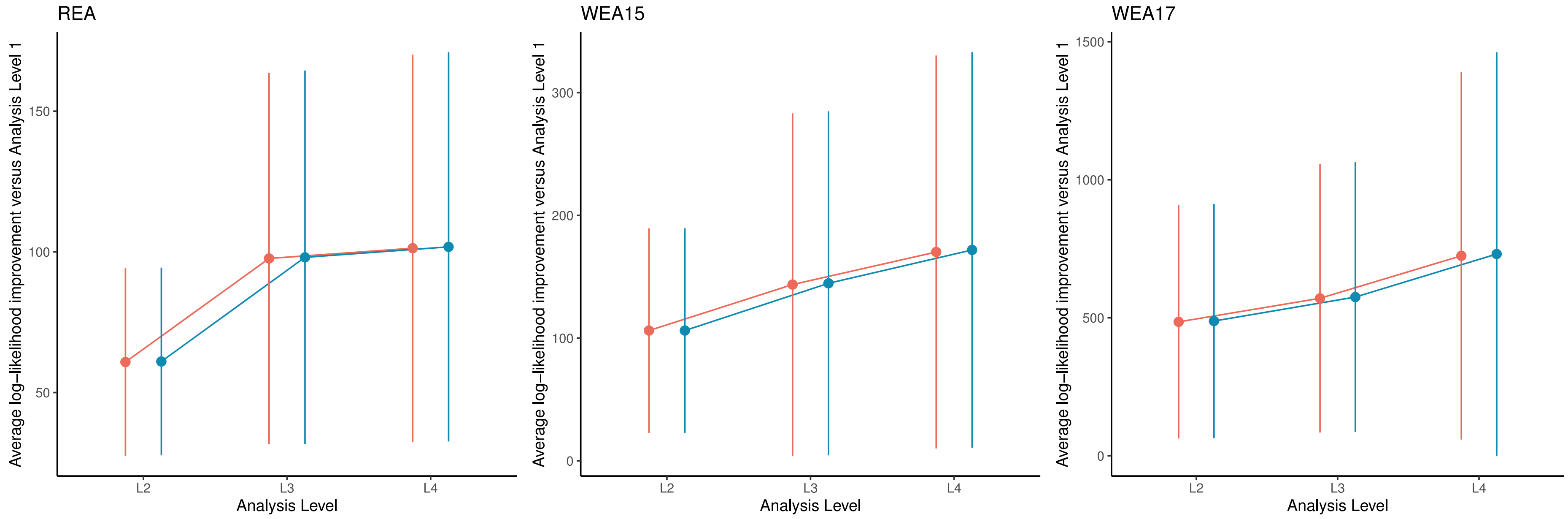

Supplementary Fig. 25. Plot of average (point: mean, bar: standard deviation) improvement in partition-specific log-likelihood over analysis level 1 from analysis levels from L2 to L4 under Ctenophora-sister (red) and Porifera-sister (blue). n = 406 gene partitions (REA dataset), 89 gene partitions (WEA15 dataset), and 31 (multi-)gene partitions (WEA17 dataset) over three comparison experiments of reanalyses and different analysis levels.

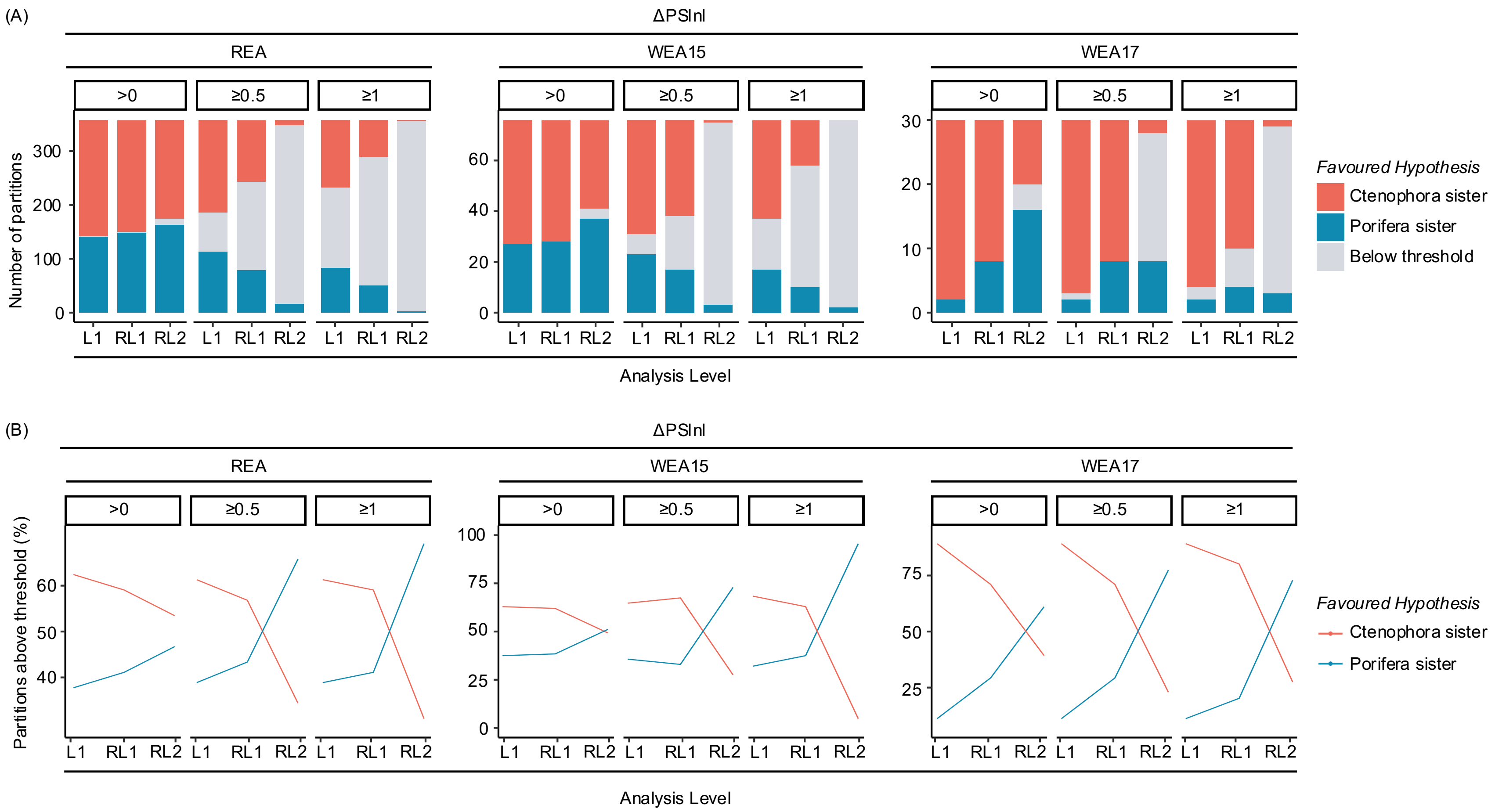

Supplementary Fig. 26. Partition-specific support for Ctenophora or Porifera sister under different analysis levels incorporating recoding. (A) Number of partitions supporting either Ctenophora or Porifera sister at L1, RL1 and RL2 at ΔPSlnl > 0, ΔPSlnl ≥ 0.5, and ΔPSlnl ≥ 1. **(B)** Relative proportion of partitions that support either Ctenophora or Porifera sister from L1-L4 (i.e. partitions that do not support either topology at that threshold level are not included).

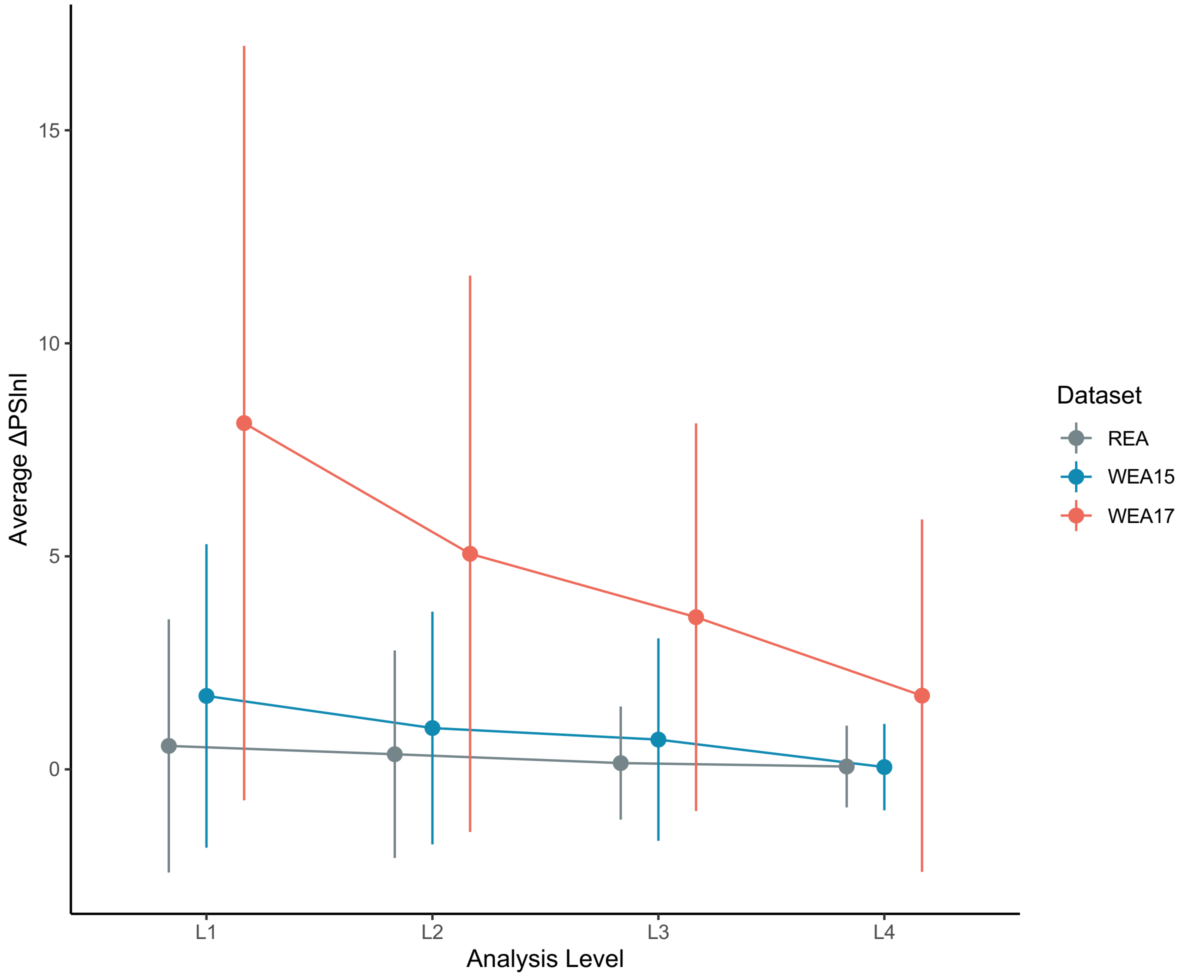

Supplementary Fig. 27. Plot of average (point: mean, bar: standard deviation) ΔPSlnl shift over analysis levels for each animal phylogeny dataset. ΔPSlnl values were calculated by subtracting the partition-specific log-likelihood values under Porifera sister from those under Ctenophora sister, meaning that positive y-axis values favour Ctenophora sister, while negative favour Porifera sister. n = 358 gene partitions (REA dataset), 76 gene partitions (WEA15 dataset), and 30 (multi-)gene partitions (WEA17 dataset) over four reanalyses at different analysis levels.

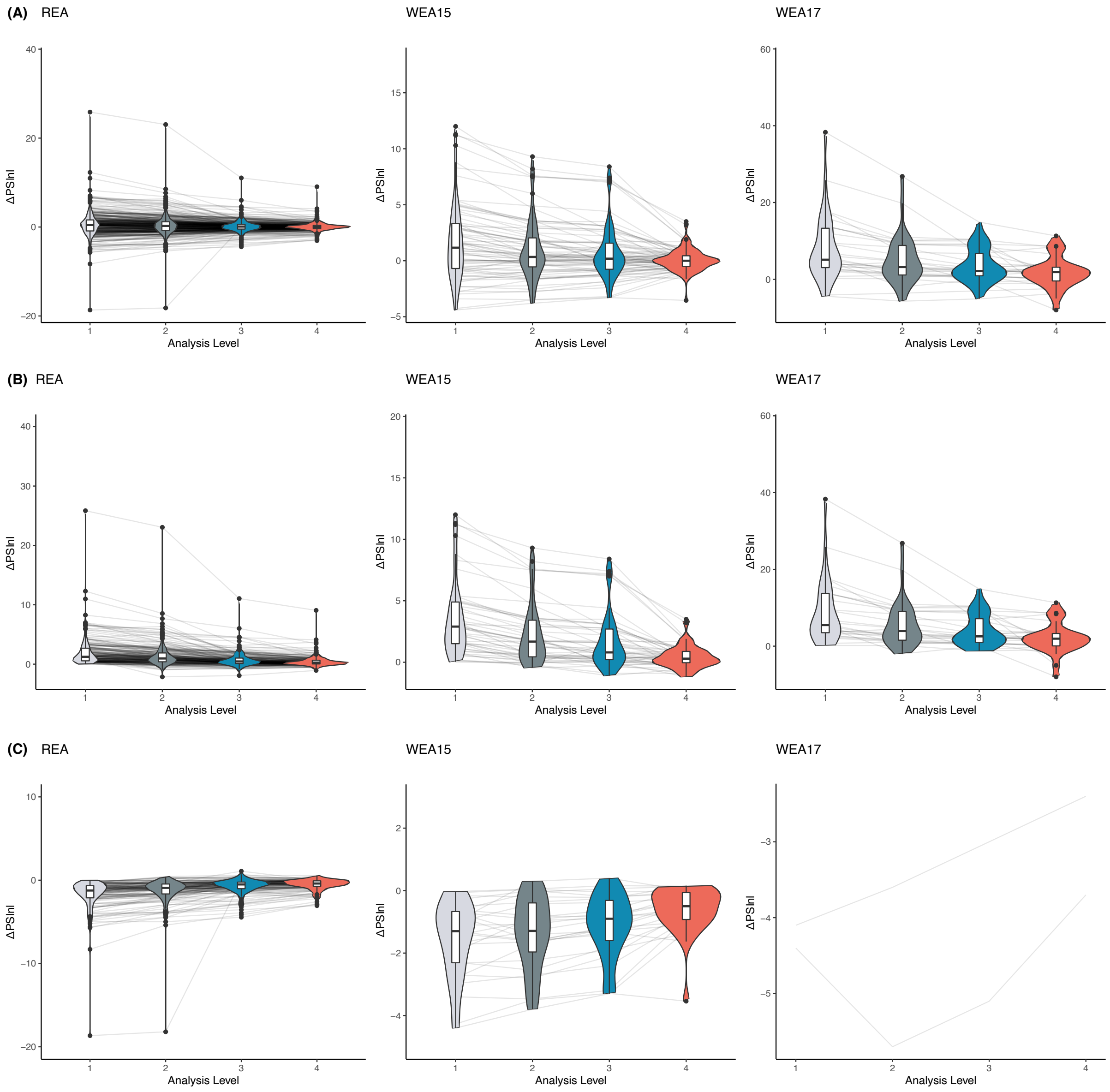

Supplementary Fig. 28. Violin plots showing the ΔPSlnl value trajectory of each partition (lines) from L1 to L4 (A), as well as separately examining those partitions that favoured either Ctenophora (B), or Porifera (C) as sister to other animals at L1. ΔPSlnl values were calculated by subtracting the partition-specific log-likelihood values under Porifera sister from those under Ctenophora sister, meaning that positive y-axis values favour Ctenophora sister, while negative favour Porifera sister. In panel (A) n = 358 gene partitions (REA dataset), 76 gene partitions (WEA15 dataset), and 30 (multi-)gene partitions (WEA17 dataset) over four reanalyses at different analysis levels. In panel (B) n = 216 gene partitions (REA dataset), 49 gene partitions (WEA15 dataset), and 28 (multi-)gene partitions (WEA17 dataset) over four reanalyses at different analysis levels. In panel (C) n = 141 gene partitions (REA dataset), 27 gene partitions (WEA15 dataset), and 2 (multi-)gene partitions (WEA17 dataset) over four reanalyses at different analysis levels. Boxplots are formed such that the middle value is the median (50% quantile), while the lower and upper hinges represent the 25% and 75% quantiles. Whiskers extend to the minima and maxima or extend to 1.5 times the interquartile range beyond the box (Tukey style) if outliers, which are drawn as individual data points, are present.

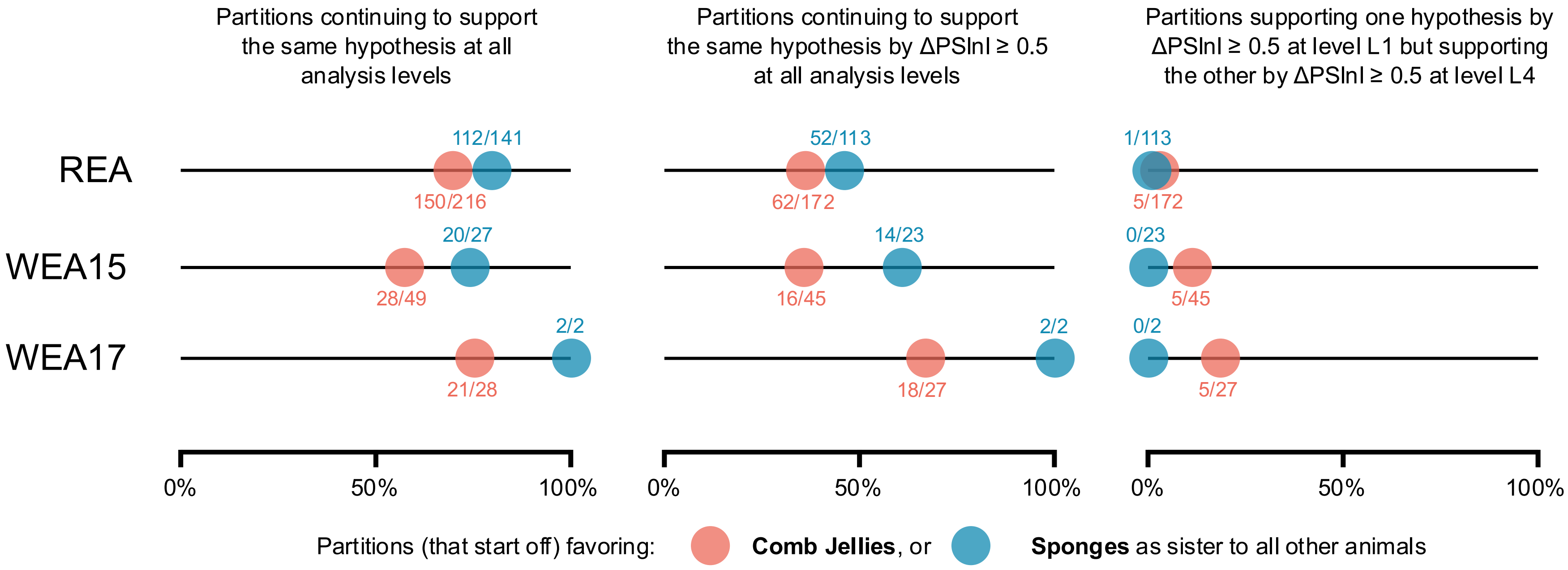

Supplementary Fig. 29. Plots showing the number and proportion of partitions that continue to support either Ctenophora or Porifera sister at all analysis levels, that continue to support either Ctenophora or Porifera sister at all analysis levels by ΔPSlnl ≥ 0.5, and that support either Ctenophora or Porifera sister at analysis level L1 by ΔPSlnl ≥ 0.5, but support the opposite hypothesis by ΔPSlnl ≥ 0.5 at analysis level L4.

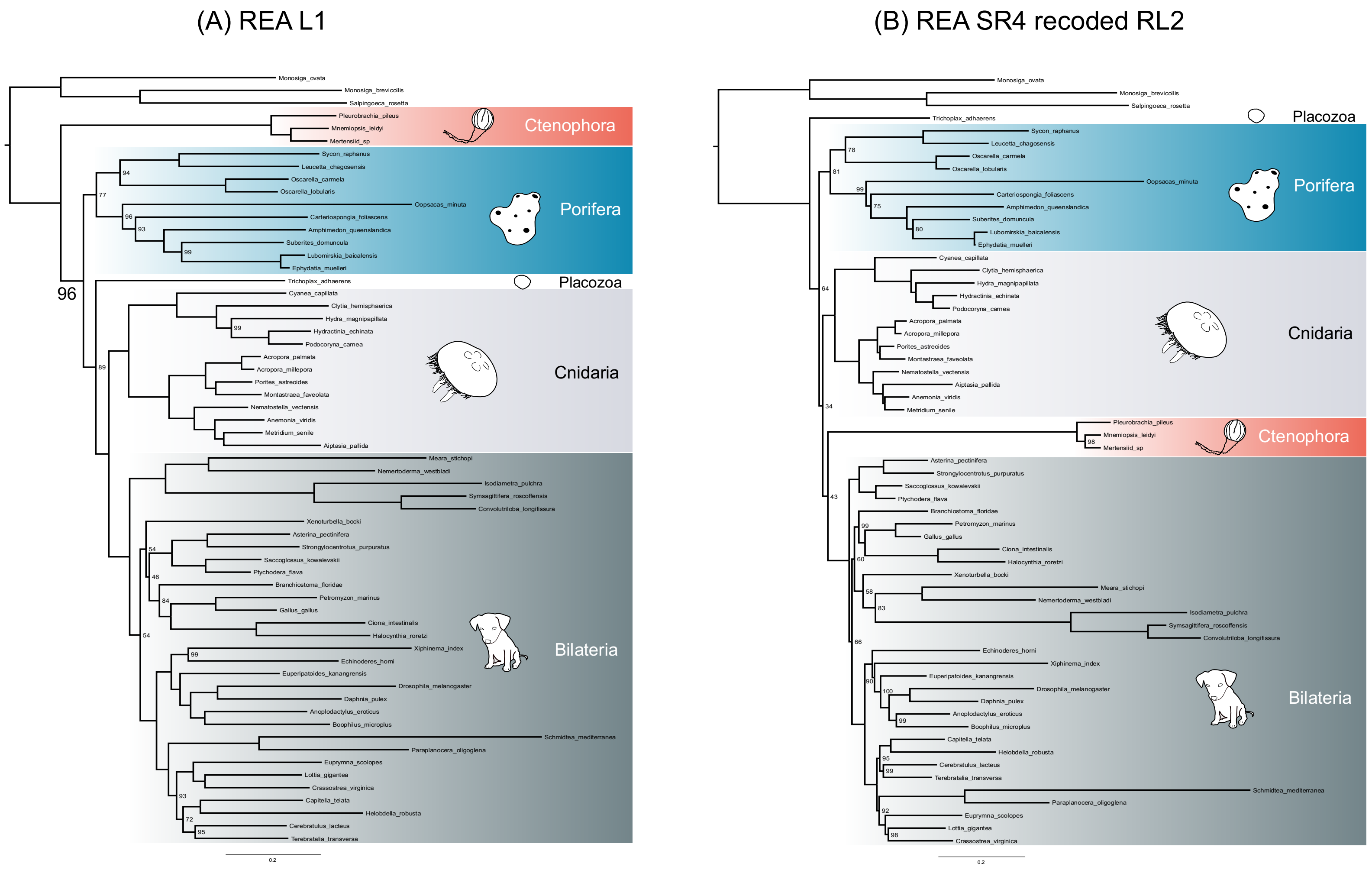

Supplementary Fig. 30. Full consensus trees with UFBOOT supports (not shown where maximal) from analyses of the REA dataset including *Xenoturbella bocki* partitioned by gene at analysis levels L1 (A) and RL2 (B).

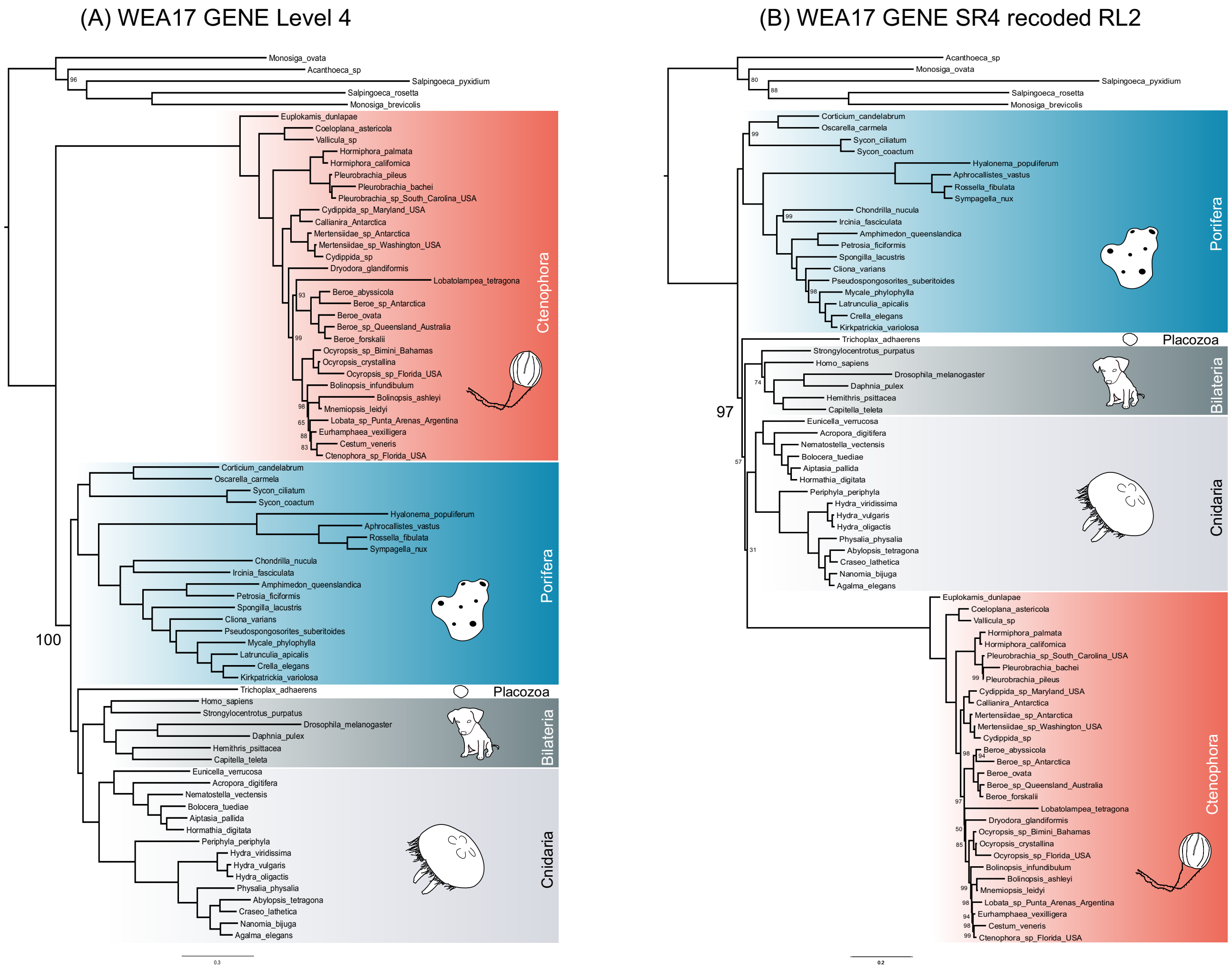

Supplementary Fig. 31. Full consensus trees with UFBOOT supports (not shown where maximal) from analyses of the WEA17 dataset partitioned by gene rather than according to the partitioning scheme from the original study at analysis levels L4 for amino acids and RL2 (A) for SR4 (B) recoded data.

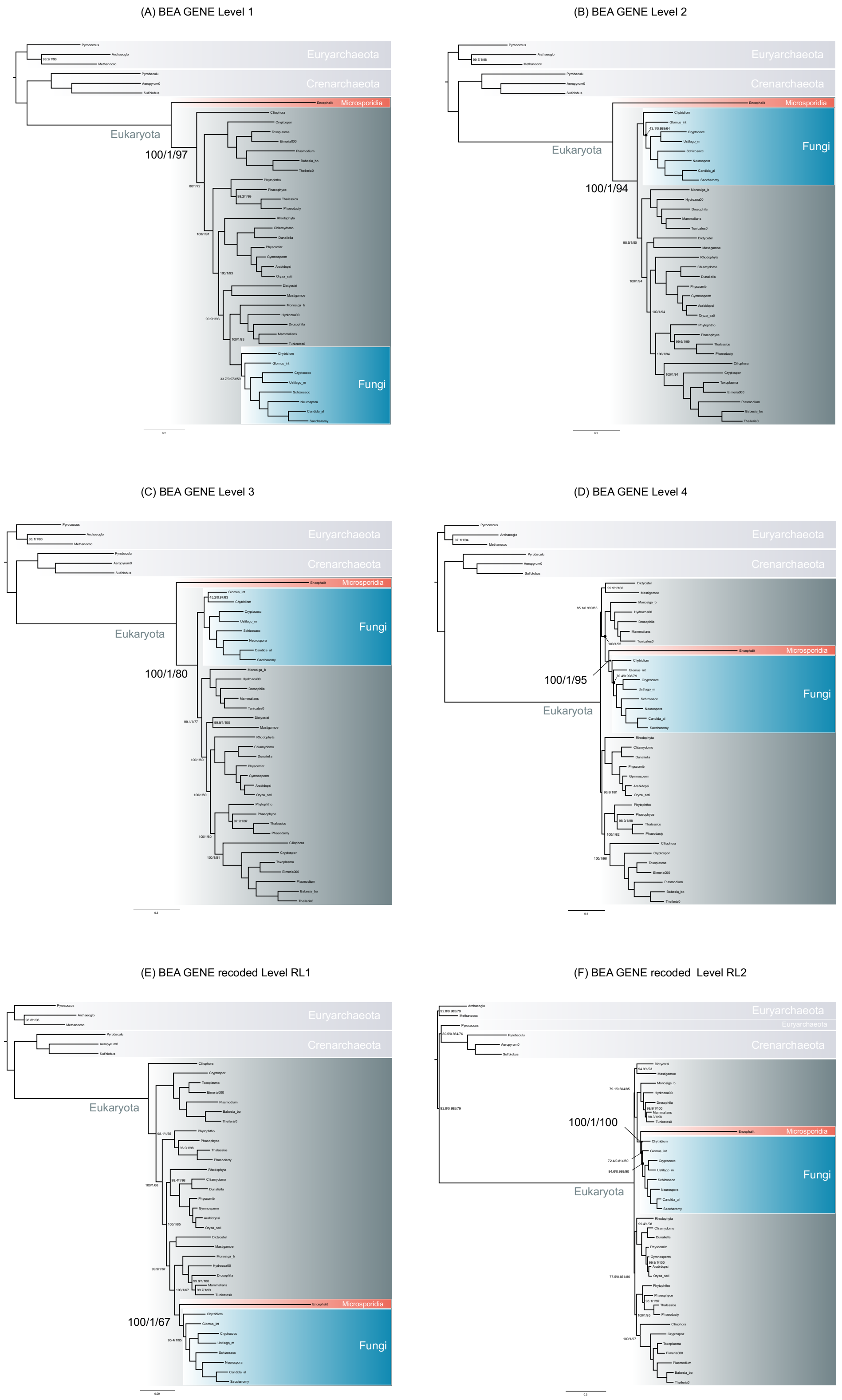

Supplementary Fig. 32. Full maximum likelihood trees with SH-aLRT/aBayes/UFBOOT supports displayed for each branch (not shown where maximal) from analyses of the BEA dataset partitioned by gene at analysis levels L1 (A), L2 (B), L3 (C), L4 (D), RL1 (E), and RL2 (F).

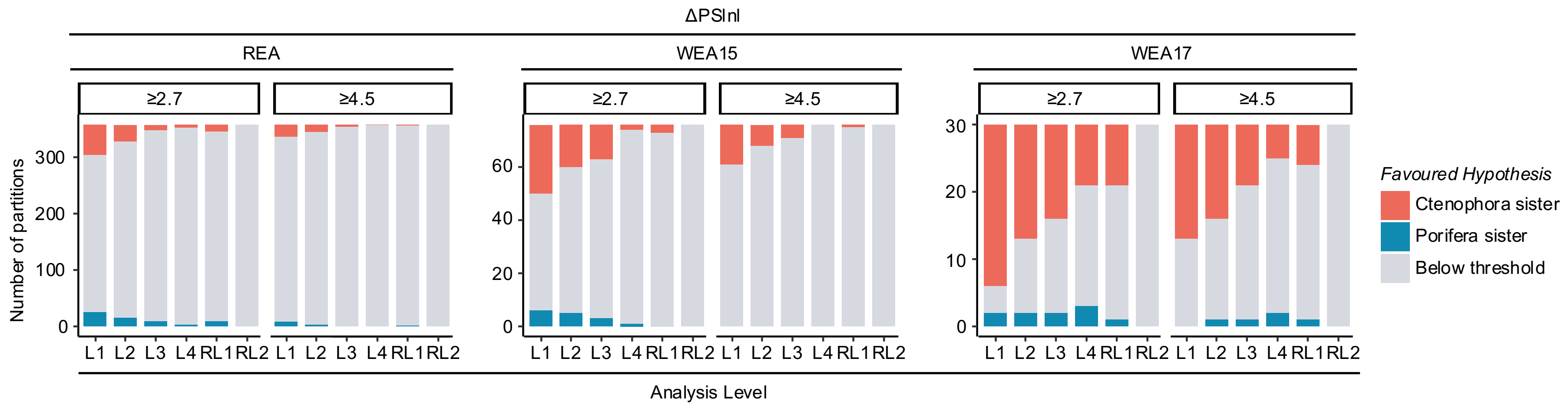

Supplementary Fig. 33. Statistically significant partition-specific support for Ctenophora or Porifera sister at all analysis levels. Number of partitions having at least ΔPSlnl ≥ 2.7 and ΔPSlnl ≥ 4.5 for either Porifera or Ctenophora sister at L1-L4, RL1 and RL2.
