# Supplementary material for: Evidence for sponges as sister to all other animals from partitioned phylogenomics with mixture models and recoding
Source: Nat Commun. 2021 Mar 19;12:1783. doi: 10.1038/s41467-021-22074-7 (PMC7979703; doi:10.1038/s41467-021-22074-7)
Supplement: Supplementary file 2 — Reporting Summary [file 41467_2021_22074_MOESM2_ESM.pdf]

nature research

Corresponding author(s): Aoife McLysaght

Last updated by author(s): Feb 7, 2021

Reporting Summary

Nature Research wishes to improve the reproducibility of the work that we publish. This form provides structure for consistency and transparency in reporting. For further information on Nature Research policies, see our Editorial Policies and the Editorial Policy Checklist.

Statistics

For all statistical analyses, confirm that the following items are present in the figure legend, table legend, main text, or Methods section.

n/a	Confirmed	
☐	☒	The exact sample size (*n*) for each experimental group/condition, given as a discrete number and unit of measurement
☐	☒	A statement on whether measurements were taken from distinct samples or whether the same sample was measured repeatedly
☒	☐	The statistical test(s) used AND whether they are one- or two-sided<br>*Only common tests should be described solely by name; describe more complex techniques in the Methods section.*
☒	☐	A description of all covariates tested
☒	☐	A description of any assumptions or corrections, such as tests of normality and adjustment for multiple comparisons
☐	☒	A full description of the statistical parameters including central tendency (e.g. means) or other basic estimates (e.g. regression coefficient) AND variation (e.g. standard deviation) or associated estimates of uncertainty (e.g. confidence intervals)
☒	☐	For null hypothesis testing, the test statistic (e.g. *F*, *t*, *r*) with confidence intervals, effect sizes, degrees of freedom and *P* value noted<br>*Give P values as exact values whenever suitable.*
☒	☐	For Bayesian analysis, information on the choice of priors and Markov chain Monte Carlo settings
☒	☐	For hierarchical and complex designs, identification of the appropriate level for tests and full reporting of outcomes
☒	☐	Estimates of effect sizes (e.g. Cohen's *d*, Pearson's *r*), indicating how they were calculated

Our web collection on statistics for biologists contains articles on many of the points above.

Software and code

Policy information about availability of computer code

Data collection	No specialist software was used for data collection
Data analysis	BLAST<br>IQtree (v. 1.5.4-omp)<br>ModelFinder (as packaged in IQtree v. 1.5.4-omp)

For manuscripts utilizing custom algorithms or software that are central to the research but not yet described in published literature, software must be made available to editors and reviewers. We strongly encourage code deposition in a community repository (e.g. GitHub). See the Nature Research guidelines for submitting code & software for further information.

Data

Policy information about availability of data

All manuscripts must include a data availability statement. This statement should provide the following information, where applicable:

- Accession codes, unique identifiers, or web links for publicly available datasets
- A list of figures that have associated raw data
- A description of any restrictions on data availability

Alignments, best-fit model and partition files, new SR4 recoded model files, and partition-specific log-likelihood value files have been uploaded to FigShare repository(https://doi.org/10.6084/m9.figshare.12746972.v1).

Field-specific reporting

Please select the one below that is the best fit for your research. If you are not sure, read the appropriate sections before making your selection.

☐ Life sciences ☐ Behavioural & social sciences ☒ Ecological, evolutionary & environmental sciences

For a reference copy of the document with all sections, see nature.com/documents/nr-reporting-summary-flat.pdf

Ecological, evolutionary & environmental sciences study design

All studies must disclose on these points even when the disclosure is negative.

Study description

We present an updated partitioned phylogenomics approach, using site-heterogeneous models and amino acid recoding, and test whether it is more robust to data heterogeneity induced errors on well characterized example datasets, before applying it to key datasets previously showing support for both comb jellies or sponges as sister to all other animals depending upon phylogenetic methodology used.

Research sample

Our analyses focused on 6 existing phylogenomic datasets from 5 previous studies.

Three of these were used as test datasets and are known to be susceptible to easily corrected long branch attraction artefacts. The first of these is a eukaryote dataset assembled by Brinkmann et al. 2005 (BEA dataset) with Archaea as the outgroup. This dataset incorrectly groups the fast-evolving Microsporidia as sister to all other eukaryotes when analysed with standard site-homogeneous models, rather than correctly grouping them with Fungi, as can be achieved using improved taxon sampling, slowly evolving sites, or site-heterogeneous models. The second and third datasets are bilaterian datasets including only Fungi as an outgroup and were generated by Lartillot et al. 2007. These two datasets are differentiated by the presence of either nematodes (LEAN dataset) or platyhelminths (LEAP). The distant fungal outgroup is known to draw the long-branching nematode or platyhelminth clades toward the tree root when using standard site-homogeneous models, while improved taxon sampling, and use of site-heterogeneous models corrects this, favoring the monophyly of Protostomia.

Three animal phylogeny datasets were selected which have been shown to support either sponges or comb jellies as sister to other animals depending on the methodology applied. The first of these is the 'EST' dataset of Ryan et al 2013 (REA dataset) which supports comb jellies as sister to other animals when using site-homogeneous models and a partitioned phylogenomics approach, but when reanalysed with the CAT model and distant outgroups removed supports sponges as sister to other animals (Pisani et al. 2015). The second is 'dataset 16' from Whelan et al. 2015 (WEA15 dataset), which was also reanalysed by Pisani et al, follows the same pattern. The third animal phylogeny dataset is 'Metazoa_Choano_RCFV_strict' from Whelan et al. 2017 (WEA17 dataset), which supports comb jelies as sister to other animals under standard site-homogeneous models, partitioning, and site-heterogeneous models, but when reanalysed pairing site-heterogeneous models with amino recoding supports sponges as sister to other animals (Feuda et al 2017).

Sampling strategy

For test datasets we collected three data that are very well characterized and have previously been shown to be susceptible to long branch attraction artefacts, but for which this can be ameliorated using site-heterogeneous models or improved taxon sampling.

For animal phylogeny datasets all three datasets were chosen because they have been analysed multiple times in the literature and have provided support for both comb jellies or sponges as sister to other animals depending upon the analysis methodology, thus they represent key datasets to resolve this debate.

Data collection

The BEA and LEA(N/P) datasets were kindly provided by Hervé Philippe and Nicolas Lartillot respectively.

The REA dataset was obtained from: https://research.nhgri.nih.gov/manuscripts/Baxevanis/science2013_supplement/.

The WEA15 dataset was obtained from: https://figshare.com/articles/Error_signal_and_the_placement_of_Ctenophora_sister_to_all_other_animals/1334306

The WEA17 dataset was obtained from: https://figshare.com/articles/Ctenophora_Phylogeny_Datasets_and_Core_Orthologues/4484138

Timing and spatial scale

All datasets were collected between 2018 and 2019.

Data exclusions

We modified two animal datasets REA and WEA15 for our analyses to include only the closest outgroups to minimize bias, and also removed the species Xenoturbella bocki from one of the REA dataset as this has previously caused problems with analyses using complex models. These modification were previously performed by Pisani et al. 2015, where they have been justified in detail. In addition we did not reanalyse the dataset of Simion et al. 2017 as, despite it being the largest dataset used to resolve the animal phylogeny yet, we judged reanalyses to be overly computationally intensive, which would also impede reproducibility.

Reproducibility

We have made all of the precise datasets, partition model files, SR4 recoded models, and partition-specific log-likelihood values from our study available for other researchers to reanalyse.

Randomization

Randomization was not relevant to this study. As our study reanalysed previously built phylogenomic datasets gene and taxon sampling were already performed. For the animal phylogeny datasets the previous studies used different dataset assembly and curation approaches, and the datasets are each made up of different (although overlapping) sets of genes and taxa.

Blinding

Blinding is not relevant to studies of this type.

Did the study involve field work? ☐ Yes ☒ No

Reporting for specific materials, systems and methods

We require information from authors about some types of materials, experimental systems and methods used in many studies. Here, indicate whether each material, system or method listed is relevant to your study. If you are not sure if a list item applies to your research, read the appropriate section before selecting a response.

Materials & experimental systems

n/a	Involved in the study
☒	☐ Antibodies
☒	☐ Eukaryotic cell lines
☒	☐ Palaeontology and archaeology
☒	☐ Animals and other organisms
☒	☐ Human research participants
☒	☐ Clinical data
☒	☐ Dual use research of concern

Methods

n/a	Involved in the study
☒	☐ ChIP-seq
☒	☐ Flow cytometry
☒	☐ MRI-based neuroimaging
